# Hepatoprotective, Antioxidant, and Anti-Hyperlipidemic Effects of Kefir Milk in High-Fat Diet-Induced Obesity: Insights from Gas Chromatography-Mass Spectrometry Profiling, Molecular Docking of Kefiran, and Liver Function Restoration

**DOI:** 10.3390/antiox14121500

**Published:** 2025-12-14

**Authors:** Imen Hammami, Sonia Ben Younes, Ridha Ben Ali, Fatma Arrari, Afef Nahdi, Michèle Véronique El May, Rym Baati, Eduardo Alberto López-Maldonado, Abada Mhamdi

**Affiliations:** 1 Laboratory of Population Health, Environmental Aggressors and Alternative Therapies (LR24ES10), Faculty of Medicine of Tunis, University of Tunis El Manar, Tunis 1007, Tunisia; benyounes_sonia@yahoo.fr (S.B.Y.); ridhabenali9@gmail.com (R.B.A.); afef.nahdi@gmail.com (A.N.); elmay_michele@yahoo.fr (M.V.E.M.); abada.mhamdi@fmt.utm.tn (A.M.); 2 Department of Basic Sciences, Histology Embryology and Cell Biology Section, Faculty of Medicine of Tunis, University of Tunis El Manar, 15 Street Djebel Lakhdar, La Rabta, Tunis 1007, Tunisia; 3 Laboratory of Functional Physiology and Valorization of Bio-Resources, Higher Institute of Biotechnology of Beja, University of Jendouba, Beja 9000, Tunisia; fatma.arrari90@gmail.com; 4 Department of Basic Sciences, Physiology Section, Faculty of Medicine of Tunis, University of Tunis El Manar, 15 Street Djebel Lakhdar, La Rabta, Tunis 1007, Tunisia; rym.baati@fmt.utm.tn; 5 Faculty of Chemical Sciences and Engineering, Autonomous University of Baja California, Tijuana 22390, Mexico

**Keywords:** antioxidant activities, high-fat diet, kefir milk, oxidative stress, hepatoprotective effects, fatty acid composition, histology tissues, molecular docking

## Abstract

The prevalence of chronic diseases, including obesity and related endocrine disorders, has risen significantly in recent decades. As a result, there has been growing interest in fermented foods with probiotic properties, such as kefir, which have potential health benefits. This study aimed to evaluate the hepatoprotective and antioxidant effects of kefir milk (KM) in a high-fat diet (HFD)-induced obesity rat model, complemented by in silico molecular docking studies with antioxidant enzymes. Twenty-four adult rats were divided into four groups: control (1 mL/100 g bw semi-skimmed cow milk), KM (1 mL/100 g bw kefir milk), HFD (1 mL/100 g bw semi-skimmed cow milk + high-fat diet), and KM/HFD (1 mL/100 g bw kefir milk + high-fat diet). After 60 days of treatment, biochemical assays and histological examinations were performed to assess the effects on lipid profiles and organ health. Kefir milk demonstrated significant antioxidant activity, with increased total phenolic content and enhanced DPPH, ABTS, and FRAP radical scavenging activities compared to commercial milk. Furthermore, KM administration protected against liver metabolic disruptions (ALT, AST, and LDH) induced by the high-fat diet and reduced lipid peroxidation in liver and testis tissues. KM supplementation also increased the activity of key antioxidant enzymes, including superoxide dismutase (SOD), catalase (CAT), and glutathione peroxidase (GPx). Additionally, KM improved the fatty acid composition and decreased the de novo lipogenesis (DNL) index, as well as enzyme activities (SCD and Elovl6) associated with the high-fat diet. Histological analysis of liver, pancreas, and heart tissues revealed that kefir milk attenuated structural damage caused by the high-fat diet, suggesting its protective role in oxidative stress regulation and organ function. These findings underscore the potential of kefir milk as a functional food for preventing metabolic disturbances and liver damage associated with obesity.

## 1. Introduction

Obesity has become a major global health crisis, with its prevalence continuing to rise, particularly in low- and middle-income countries [1,2]. According to projections by the World Obesity Federation (WOF), approximately one billion people worldwide will live with obesity by 2030, including one in five women and one in seven men [2]. Between 1980 and 2015, global obesity rates more than doubled, with notable increases in both children (5%) and adults (12%) [3]. In OECD (Organization for Economic Cooperation and Development) countries, the average obesity rate is currently 19.5%, ranging from 3.7% in Japan to 38.2% in the United States [4]. This growing trend is strongly associated with various metabolic comorbidities, such as type 2 diabetes, non-alcoholic fatty liver disease (NAFLD), hyperlipidemia, and metabolic syndrome [5,6]. In response to this public health challenge, dietary interventions such as the consumption of kefir have been proposed to reduce the risk of metabolic diseases [7]. Kefir is a mildly alcoholic, fermented beverage produced by fermenting kefir grains in milk or water [8]. Originating in the Balkans, Eastern Europe, and the Caucasus, kefir’s consumption has spread globally due to its recognized health benefits [9]. It is widely consumed in Eastern Europe, where it is industrially produced, and in countries such as Argentina, Taiwan, Portugal, Turkey, and France [10]. In Tunisia, kefir is traditionally made on a small scale, with grains often shared among individuals who consume it for its health-promoting properties.

Kefir contains a unique combination of exopolysaccharides, proteins, and kefiran, a polysaccharide produced by kefir grains during fermentation. Kefiran, made up primarily of glucose and galactose, contributes to kefir’s gel-like texture [11]. It is known for its antioxidant, antimicrobial, and immunomodulatory effects [12]. Historically, kefir has been used as a remedy for various ailments, such as tuberculosis, cancer, and gastrointestinal disorders, especially when modern medical treatments were unavailable [13]. Today, kefir is widely recognized as a probiotic beverage with significant health-promoting potential. It is considered easy to produce, safe, and cost-effective [14]. According to the Food and Agriculture Organization (FAO) and the World Health Organization (WHO), probiotics are live microorganisms that confer health benefits when consumed in adequate amounts. The bioactive compounds in kefir, particularly kefiran, have shown notable physicochemical properties and biological activities [15]. Numerous studies have demonstrated the health benefits of kefir, including antimicrobial, antitumor, hypocholesterolemic, antihypertensive, antidiabetic, and immunomodulatory effects, and improved lactose digestion [9]. These effects are attributed to the interactions between kefir’s microorganisms and their metabolic products during fermentation [8]. However, despite the growing body of research on the potential of kefir in preventing obesity and associated metabolic disorders, the field remains in its early stages, with a notable lack of human clinical trials. Additionally, there is significant variability in the production processes used for kefir in these studies. To address these gaps, the present study builds upon recent work conducted in our laboratory, where we examined the lipid and metabolic profiles of rats fed a hyperlipidemic diet and evaluated the protective effects of kefir milk consumption, supported by biochemical and histopathological analyses. Moreover, in silico molecular docking studies were performed on kefiran, the major bioactive molecule in kefir milk, in interaction with antioxidant enzymes such as superoxide dismutase (SOD) (PDB: 4 cm), catalase (CAT) (PDB: 1 dgb), glutathione peroxidase (GPx) (PDB: 3 kij), and NADPH oxidase (PDB: 2 CDU), to further explore its potential as an antioxidant agent.

This study offers a novel, multidisciplinary approach combining in-depth biochemical and histological analyses with in silico studies to investigate the interactions between kefir and key antioxidant enzymes. The use of molecular simulations complements experimental analyses and provides a mechanistic understanding of kefir’s effects at the molecular level, making a significant contribution to the existing literature. To our knowledge, few studies have integrated such simulations to explore kefir’s ability to modulate enzymatic activities and neutralize free radicals. The main hypothesis of our study is that kefir offers hepatic and antioxidant protection against damage induced by a high-fat diet. Our in silico findings support these interactions and further validate our experimental results. The combined approach of molecular simulations and biochemical assessments provides a deeper understanding of the molecular mechanisms through which kefir exerts its protective effects.

## 2. Materials and Methods

### 2.1. Kefir Milk Preparation

The kefir grains were obtained from the local market of traditional medicinal plants (Souk el Attarin, Tunis, Tunisia). The method of kefir milk preparation is described in the study of Hammami et al. (2022) [16]. In accordance with the traditional method, the kefir culture was as follows: (10% *w*/*v*), 10 g of kefir grains were inoculated into 100 mL of commercial liquid pasteurized semi-skimmed cow milk (Delice, Tunis, Tunisia). The mixture was cultivated sequentially at 23 °C for 12 h and filtered. The grains were removed, and the kefir was stored at 4 °C. The beverage was given fresh to the rats immediately after the fermentation period.

The physicochemical analysis of the kefir milk was as follows: pH (22 °C) = 4.07, acidity (D°) = 88, fat (g/L) = 18.63, dry matter (g/L) = 11.11, density (22 °C) = 1.025, lactose (g/L) = 34, and proteins (g/L) = 28.04 [16].

### 2.2. Determination of the Total Antioxidant Capacity (TAC) of Commercial Milk and Kefir Milk

#### 2.2.1. Free Radical Scavenging Activity on DPPH

The antioxidant activity was evaluated using the 2,2-diphenyl-1-picrylhydrazyl (DPPH) radical scavenging assay, following the method described by Liu et al. (2005) [17]. In summary, varying volumes (10–100 µL) of commercial milk or kefir milk were added to 0.5 mL of a methanolic solution containing DPPH radicals, resulting in a final concentration of 0.2 mM DPPH. The mixture was thoroughly combined and kept in the dark for 30 min. Absorbance was then measured at 517 nm, and the percentage of DPPH radical scavenging activity was determined using the formula: (%) = [1 − (absorbance of sample/absorbance of control)] × 100.

#### 2.2.2. ABTS Assay

The ABTS assay evaluates the capacity of antioxidant compounds to neutralize ABTS (2,2′-azino-bis (3-ethylbenzothiazoline-6-sulfonic acid)) radicals in an aqueous medium. The antioxidant activities of commercial milk and kefir milk were assessed using the ABTS^+^ radical cation decolorization method [18]. ABTS^+^ radicals were produced by reacting ABTS (7 mM in water) with potassium persulfate (2.45 mM) in a 1:1 (*v*/*v*) ratio and storing the mixture in the dark at room temperature for 12–16 h. The ABTS^+^ stock solution was diluted with ethanol to create a working solution with an absorbance of 0.700 at 734 nm. Subsequently, 10–100 µL of commercial milk or kefir milk was added to 1 mL of the diluted ABTS^+^ working solution, mixed thoroughly, and the absorbance was measured at 734 nm after 30 min, using a blank as a reference. The percentage of ABTS+ radical scavenging activity was calculated as: (%) = [1 − (absorbance of sample/absorbance of control)] × 100.

#### 2.2.3. Determination of Total Phenol Content (TPC)

The total phenolic content was assessed using the method described by Ellafi et al. (2023) [19]. In summary, 10–100 μL of either commercial milk or kefir milk was mixed with 1 mL of Folin–Ciocalteu reagent and incubated for 5 min in the dark. Subsequently, 0.8 mL of a 7% Na_2_CO_3_ solution was added, and the reaction mixture was left in darkness for 30 min. The absorbance was recorded at 727 nm. The phenolic content was calculated using a gallic acid standard curve (50–200 μg/mL) and expressed as milligrams of gallic acid equivalents per 100 mL of milk.

#### 2.2.4. Ferric Reducing Antioxidant Power Assay

The ferric reducing antioxidant power (FRAP) assay was conducted following the protocol of Liu et al. (2005) [17]. In a dry test tube, 0.01–0.1 mL of commercial milk or kefir milk was combined with 450 μL of distilled water and 50 μL of FeCl_2_ in a dry test tube. After a 5 min incubation, 200 μL of ferrozine solution (5 mM) was added. The reaction mixture was incubated for 10 min, and absorbance was measured at 562 nm. For blanks, distilled water replaced ferrozine, while the control contained distilled water instead of the sample. EDTA served as the reference. The ferrous ion chelating activity was calculated as (%) = [1 − (absorbance of sample)/(absorbance of control)] × 100, with higher percentages indicating stronger chelating activity.

### 2.3. Animals and Study Design

The following protocol was based on a previous study [16]. Twenty-four adult male rats were obtained from the Pasteur Institute of Tunis. After a seven-day period of physical and behavioral acclimatization to the new environment, the rats were randomly assigned to one of four groups, with six rats per group (three rats per cage):

- Control Group (C): These rats were fed a standard diet (ND) and received 1 mL/100 g of semi-skimmed cow milk through an intragastric administration.
- Kefir Group (KM): The rats were given a normal diet (ND) and administered 1 mL/100 g of body weight of kefir milk intragastrically.
- High-Fat Diet Group (HFD): These rats were placed on a high-fat diet and received 1 mL/100 g of body weight of semi-skimmed cow milk intragastrically.
- Kefir + High-Fat Diet Group (KM/HFD): The rats were given a high-fat diet and administered 1 mL/100 g of body weight of kefir milk through intragastric delivery.

The high-fat diet was prepared according to the protocol described by Smine S et al. 2017 [20]. The standard rat chow, in pellet form, consisted of 3% fat, 40% carbohydrates, and 14.5% protein. To prepare the high-fat diet (HFD), commercial pellets were soaked in warmed liquid fat derived from animal sources (sheep) for 15 min, then allowed to dry at room temperature. This high-fat diet, containing 28% fat, 32% carbohydrates, and 11.6% proteins, was fed to the rats for a period of 60 days to induce obesity.

All the treatments were given in the morning (between 08:30 a.m. and 09:30 a.m.). After 60 days of treatment, the rats were anesthetized with 80 mg/kg i.p. ketamine. Blood from the brachial artery was immediately collected in heparinized tubes and centrifuged at 3000× *g* for 10 min at +4 °C for biochemical assays. The abdominal cavity of each animal was opened, and the liver, pancreas, kidney, and fat around the epididymis, pancreas, liver, and kidney tissues were collected and weighed. For each animal, the liver, pancreas, and kidney were fixed in 10% formaldehyde, followed by routine paraffin embedding for histological analysis. The experimental protocol and all procedures involving animals were authorized by the Ethics Committee of the Faculty of Medicine in Tunis [16].

### 2.4. Functional and Metabolic Parameters

Organ function was assessed by measuring the activities of ALT, AST, LDH, total bilirubin, creatinine, total cholesterol, triglycerides, glucose, and total protein using a Beckman automated system (Unicel DXC800 Synchron Clinical System, Beckman Coulter, Brea, CA, USA) at the National Institute of Nutrition and Food Technology (Tunis, Tunisia).

### 2.5. Analysis of Oxidant Status in the Liver and Testis

Each tissue (liver and testis) was homogenized in 2 mL of phosphate-buffered saline (pH 7.2) and then centrifuged at 4600 rpm for 10 min. The supernatant was collected for the analysis of oxidative stress biomarkers.

#### 2.5.1. Lipid Peroxidation (LPO) Measurement

LPO was quantified by measuring MDA using the double heating method [21]. In brief, 0.2 g of testis homogenate was mixed with a BHT-TCA solution (1% BHT in 20% TCA), followed by centrifugation at 1000× *g* for 5 min at 4 °C. The supernatant was combined with a mixture of 0.5 N HCl and 120 mM TBA in 26 mM Tris and heated at 80 °C for 10 min. After cooling, the absorbance of the resulting chromophore was measured at 532 nm using a UV-vis spectrophotometer (Labomed, UV-2650). MDA levels were calculated based on the extinction coefficient of the MDA-TBA complex (1.56 × 10 ^5^ M ^−1^ cm ^−1^), and lipid peroxidation was expressed as nmol of MDA per mg of protein.

#### 2.5.2. Thiol Group Measurements

The total concentration of thiol groups (−SH) was measured using the methods of Boyne AF and Ellman GL (1972) [22]. Briefly, liver tissue aliquots were mixed with 100 μL of 10% SDS and 800 μL of 10 mM phosphate buffer (pH 8). The absorbance was recorded at 412 nm (A0). Then, 100 μL of DTNB was added, and the mixture was incubated at 37 °C for 60 min. After incubation, the absorbance was measured again at 412 nm (A1). The thiol group concentration was calculated by subtracting A0 from A1 and using the molar extinction coefficient of 13.6 × 10^3^ M⁻^1^ cm⁻^1^. Results were expressed as nmol of thiol groups per mg of protein.

#### 2.5.3. Antioxidant Enzyme Activity Assay

Superoxide dismutase (SOD) activity was assessed using a modified epinephrine assay [23]. At an alkaline pH, the superoxide anion (O2−) promotes the autoxidation of epinephrine to adenochrome. SOD inhibits this reaction, thus reducing adenochrome formation. One SOD unit is defined as the amount of enzyme that decreases adenochrome formation by 50%. The enzyme extract was mixed with a 2 mL reaction solution containing 10 μL of bovine catalase (0.4 U/μL), 20 μL of epinephrine (5 mg/mL), and 62.5 mM sodium carbonate/bicarbonate buffer (pH 10.2). Absorbance changes were measured at 480 nm. SOD activity was calculated as units per milligram of protein.

Catalase (CAT) activity was measured by monitoring the initial rate of H_2_O_2_ degradation at 240 nm [24]. The reaction mixture consisted of 30 mM H_2_O_2_ in 50 mM phosphate buffer (pH 7.0). CAT activity was calculated using the extinction coefficient of 40 mM^−1^ cm^−1^ for H_2_O_2_ and expressed as nanomoles of H_2_O_2_ consumed per minute per milligram of protein.

Glutathione peroxidase (GPx) activity was determined following the method of Flohé and Günzler WA (1984) [25]. The reaction mixture consisted of 0.2 mL of liver supernatant, 0.4 mL of 5 mM H_2_O_2_, 0.2 mL of 0.1 M phosphate buffer (pH 7.4), and 0.2 mL of 4 mM glutathione (GSH). The mixture was incubated at 37 °C, and the reaction was halted by adding 0.5 mL of 5% (*w*/*v*) trichloroacetic acid (TCA). After centrifugation at 1500× *g* for 5 min, a 0.2 mL aliquot of the supernatant was combined with 0.5 mL of 10 mM DTNB and 0.5 mL of 0.1 M phosphate buffer (pH 7.4). Absorbance was measured at 412 nm. GPx activity was calculated as nmol of GSH consumed per minute per milligram of protein.

#### 2.5.4. Protein Determination

Protein concentration was measured using the Bradford assay [26], with serum albumin serving as the standard.

### 2.6. Gas Chromatography—Mass Spectrometry (GC—MS) Analysis

#### 2.6.1. GC—MS Protocol

The visceral white adipose tissue surrounding the abdominal and pelvic cavities was collected for GC-MS analysis [27]. Fatty acids were extracted by homogenizing and sonicating 100 mg of adipose tissue in a chloroform-methanol solution (2:1) for 10 min, followed by the addition of 1 mL of 15% NaCl and centrifugation. The organic phase was evaporated, and the residue was dissolved in 20 µL of hexane (Sigma-Aldrich, St. Louis, MO, USA). Fatty acid separation and identification were conducted according to the ISO 5509 method using a gas chromatograph (6890 N, Agilent Technologies, Santa Clara, CA, USA) equipped with a flame ionization detector and HP-Innowax capillary column (30 m × 0.32 mm × 0.25 µm). One microliter of each sample was injected with nitrogen as the carrier gas at a constant flow of 1.0 mL/min and a split ratio of 50:1. The injector and detector temperatures were set at 230 °C and 280 °C, respectively. The column temperature program included an initial temperature of 150 °C for 1 min, a ramp to 210 °C at 15 °C/min (held for 5 min), and a further increase to 250 °C at 5 °C/min, maintained until the 25 min analysis completion. Fatty acids were identified by comparison with standards (Sigma) and quantified as percentages of the total fatty acid peak areas [27].

#### 2.6.2. Elongation and Desaturation Indices

Indices were calculated by determining the ratios of specific products to their respective substrates. The elongation index for 16:0 (EI16) was defined as the ratio of (18:0+18:1n-9) to 16:0, while the elongation index for 16:1n-7 (EI16:1n-7) was defined as the ratio of 18:1n-7 to 16:1n-7. Additionally, the total desaturation index (TDI) was calculated as the ratio of monounsaturated fatty acids (MUFAs: 16:1n-7, 18:1n-7, 18:1n-9) to saturated fatty acids (SFAs: 14:0, 16:0, 18:0) [28].

### 2.7. Histological Analysis

To assess the morphological changes within the liver, pancreas, testis, and heart tissues, organs from each rat were extracted and fixed in formaldehyde solution (10%). The tissues were passed through increasing concentrations of ethanol, cleared in toluene, and then embedded in paraffin. Four-micron-thick sections were subsequently prepared and stained with hematoxylin and eosin. The tissues were analyzed under a light microscope, and the degree of histological injury was determined. The same histological technique was used to observe the structure of the kefir grains used in our study.

### 2.8. In Silico Study

We used molecular docking, a potent computational method in structure-based drug discovery, to explore the potential binding interactions of the major compounds of kefir milk, such as kefiran, with the catalytic site of the targets and to clarify the associated inhibitory impact. Kefir is a beverage produced from milk or water that contains bacteria and polysaccharides called kefir grains [29]. Equal amounts of galactose and glucose make up the polysaccharides found in milk kefir grains, also referred to as kefiran. Its synthesis is correlated with the presence of Lactobacillus kefiranofaciens and Lactobacillus kefiri in kefir grains and is responsible for the binding of microorganisms within the grains [30]. First, three protein structures, SOD (PDB: 4 mcm), CAT (PDB: 1 dgb), GPx (PDB: 3 kij), and NADPH oxidase (PDB: 2 CDU), were downloaded from the RCSB Protein Data Bank (PDB), and the chemical structures of kefiran were acquired from PubChem. The SOD, CAT, GPx, and NADPH oxidase structures were selected to study the antioxidant properties of the kefiran compounds. The target macromolecules were processed with AutoDock 4.2 software. This method eliminates all attached ligands and water molecules from the targets, adding polar hydrogens and Kollman charges [31]. The docking procedure against SOD, CAT, and GPx employed a grid box centered on the protein with 60 × 60 × 60 dimensions and 0.375 Å spacing or NADPH oxidase with 40 × 40 × 40 dimensions and 0.375 Å spacing. The values of the grid center coordinates (x, y, z) were as follows: 12.03, 126.24, and 13.85 for SOD; 20.19, 36.56, and 52.14 for CAT; 19.43, −6.93, and 20.30 for GPx; and 4.90, 5.24, and 4.65 for NADPH oxidase. The strongest interactions of the ligand—receptor complexes’ two- and three-dimensional interactions were displayed via the Discovery Studio 2021 program [32].

### 2.9. Statistical Analysis

Statistical analysis was performed via BIOSTAT software. All values are reported as the mean ± standard deviation (SD). The difference between all groups was estimated via the Kruskal—Wallis test. For multiple comparisons, the Mann—Whitney U test was used, with statistical significance at *p* < 0.05. Additionally, a correlation test was performed to compare the hepatic tissue and testicular tissue.

## 3. Results

### 3.1. Antioxidant Capacity and Total Phenol Contents of Commercial Milk and Kefir Milk

Table 1 shows that the total phenolic compound (TPC) content of commercial milk (65 mg GAE/100 mL of milk) increased after fermentation (97.05 mg GAE/100 mL of milk). The total antioxidant capacity (TAC) of commercial milk and kefir milk was evaluated via three methods: DPPH radical scavenging, ABTS radical scavenging, and iron-reducing antioxidant potential. The results revealed significant differences in the antioxidant capacity between commercial milk and kefir milk (Table 1).

The DPPH and ABTS values were lower in commercial milk (3.2 mg/100 mL, 15 mg/100 mL) than in kefir milk (4.2 mg/100 mL, 25 mg/100 mL), whereas the FRAP values were higher in commercial milk (1.29 mg/100 mL) than in kefir milk (0.24 mg/100 mL). Compared with the DPPH and FRAP methods, the ABTS assay yielded higher values for both commercial and kefir milk.

### 3.2. Effect of Kefir Milk on Body Weight (bw) and Fat and Liver Relative Weights

The data in Table 2 show that kefir milk consumption induced a significant decrease in bw (*p* = 0.04) in the KM group compared with the control group (C). The same observation was reported in the KM/HFD group compared with the HFD group (*p* = 0.03). Our findings revealed a significant decrease in bw in the KM and KM/HFD rats compared with the HFD-fed rats. These results were confirmed by the significant decrease (*p*= 0.03) in the relative fat weights in the KM and KM/HFD groups compared with those in the HFD group. A highly significant increase (*p* = 0.003) in relative fat weight was reported in the HFD group (4.85 g ± 0.47) compared with the control group (2.58 g ± 0.50). There was no significant difference in the relative weight of the liver among the treatment groups (*p* > 0.05).

### 3.3. Effect of Kefir Milk on Liver Metabolic Parameters

Table 3 shows the liver levels of metabolic parameters. The rats that consumed kefir milk (KM) presented significantly lower total cholesterol (TC) (13.97%, *p* = 0.02) and total triglyceride (TG) (21.68%, *p* = 0.02) levels than did the control group (C). In contrast, the results of rats fed a high-fat diet (HFD) revealed significant increases in total cholesterol (TC), triglyceride (TG), and glucose levels of 26.30% (*p* = 0.03) and 16.31% (*p* = 0.02), respectively. In parallel, the total protein level decreased significantly, by 26.29% (*p* = 0.02), compared with that in the control group. Importantly, compared with those in the HFD group, the daily consumption of kefir milk in the KM/HFD group resulted in significant decreases in TC (22.45%, *p* = 0.02) and TG (17.91%, *p* = 0.04) levels.

### 3.4. Effect of Kefir Milk on Liver and Kidney Functions

Compared with the control group, the HFD group presented significant increases in ALT (119.66 ± 10.66, *p* = 0.04), AST (182.74 ± 19.36, *p* = 0.04), and LDH (661.33 ± 105.57, *p* = 0.03) activities (Table 4). The consumption of kefir milk at a 1 mL/100 g bw dose significantly protected against the disturbance of metabolic parameters induced by a high-fat diet. Compared with the control, the serum bilirubin and creatinine levels were not significantly different (Table 4).

### 3.5. Oxidative Stress Status in the Liver and Testis

#### 3.5.1. Effect of Kefir Milk on Lipid Peroxidation and Thiol Group Content

**Figure 1:**
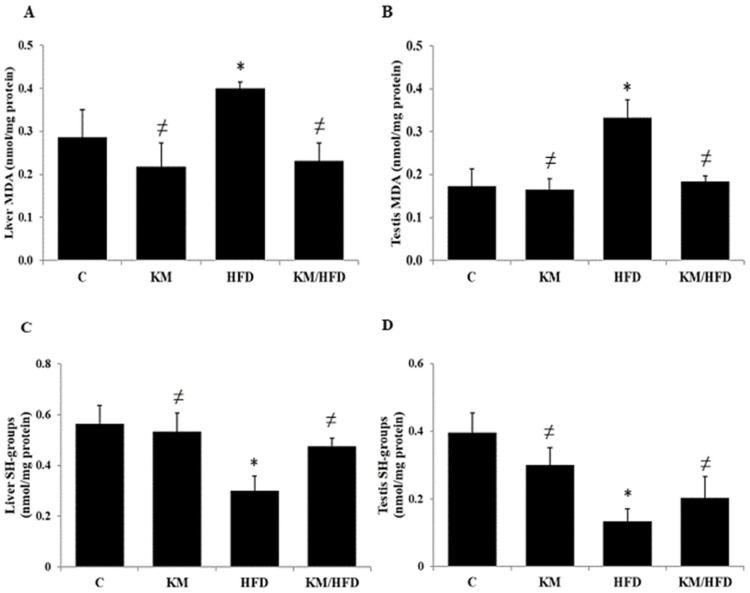
Chronic effect of kefir milk on high-fat diet-induced changes in liver (**A**,**C**) and testis (**B**,**D**) MDA and (-SH) group levels. (C): control rats consumed a normal diet; (KM): rats consumed a normal diet + 1 mL/100 g bw of kefir milk; (HFD): rats consumed a high-fat diet; (KM/HFD): rats consumed a high-fat diet + 1 mL/100 g bw of kefir milk. All parameters were expressed as nmol/mg of protein in liver and testis organs. Data are expressed as mean values (*n* = 6). The asterisk (*) indicates significant differences (*p* < 0.05) compared to control rats. The hash symbol (≠) indicates significant differences (*p* < 0.05) compared to HFD rats.

Compared with the control diet (C), the high-fat diet (HFD) significantly increased the liver (0.40 nmol/mg protein vs. 0.29 nmol/mg protein, *p* = 0.04) and testis (0.33 nmol/mg protein vs. 0.17 nmol/mg protein, *p* = 0.02) MDA levels, an index of lipid peroxidation (LPO) (Figure 1A,B). In parallel, we noted a significant decrease in the liver thiol (-SH) group compared with the (C) group (0.33 nmol/mg protein vs. 0.52 nmol/mg protein, *p* = 0.02) (Figure 1C). In the testis, we noted the same modification (0.12 nmol/mg protein vs. 0.29 nmol/mg protein, *p* = 0.02) (Figure 1D). The consumption of kefir milk (1 mL/100 g bw) significantly protected against LPO as well as the decrease in -SH group levels induced by a high-fat diet. These observations were also found in the KM/HFD group (Figure 1A–D).

A significant positive correlation was observed between MDA levels in the liver and testes (*n* = 24, r = 0.809, *p* < 0.05). These results indicate a strong association between oxidative stress markers in these two tissues. In addition, a significant correlation was observed between thiol group (SH-group) levels in the liver and testes (*n* = 24, r = 0.793, *p* < 0.05). These results confirm a strong association between oxidative stress markers in these two tissues (see Supplementary Materials).

#### 3.5.2. Effect of Kefir Milk on Antioxidant Enzyme Activities

**Figure 2:**
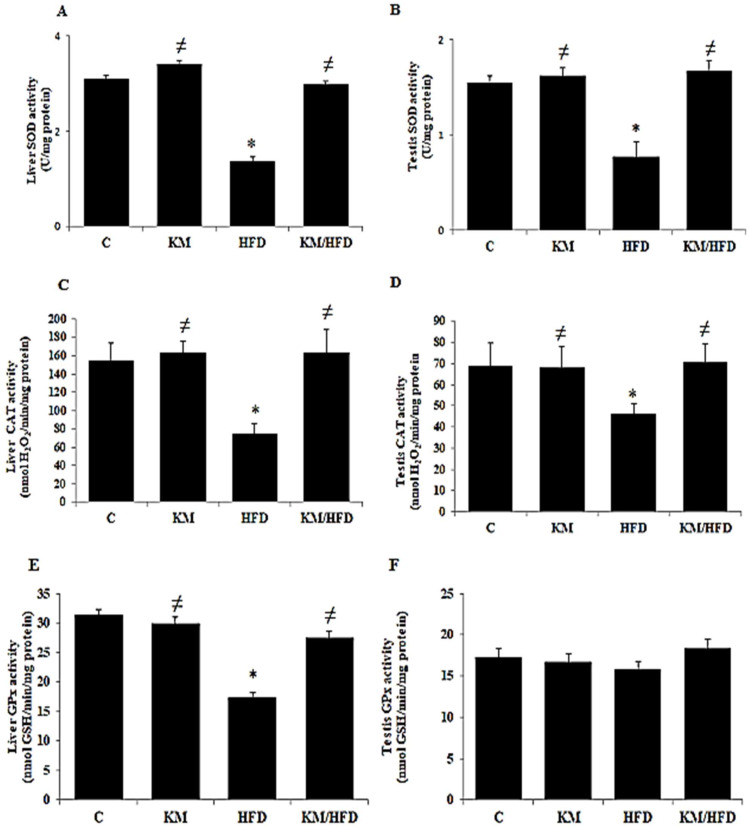
Chronic effect of kefir milk on high-fat diet-induced changes in liver (**A**,**C**,**E**) and testis (**B**,**D**,**F**) SOD, CAT, and GPx activities. (C): control rats consumed a normal diet; (KM): rats consumed a normal diet + 1 mL/100 g bw of kefir milk; (HFD): rats consumed a high-fat diet; (KM/HFD): rats consumed a high-fat diet + 1 mL/100 g bw of kefir milk. All parameters were expressed as nmol/min/mg of protein. Data are expressed as mean values (*n* = 6). The asterisk (*) indicates significant differences (*p* < 0.05) compared to control rats. The hash symbol (≠) indicates significant differences (*p* < 0.05) compared to HFD rats.

The effects of kefir milk on antioxidant enzyme activities in rats that consumed a high-fat diet are summarized in Figure 2. A high-fat diet (HFD) induced a significant increase in liver SOD activity compared with that of the control group (6.36 ± 0.76 U/mg protein vs. 3.09 ± 0.79 U/mg protein, *p* = 0.02) (Figure 2A). The same increase was observed in the testis (3.44 ± 0.40 U/mg protein vs. 1.54 ± 0.07 U/mg protein, *p* = 0.03) (Figure 2B). The consumption of kefir milk significantly reversed this increase in the KM (3.40 ± 0.69 U/mg protein, *p* = 0.02) and KM/HFD (3.82 ± 0.58 U/mg protein, *p* = 0.02) groups compared with the HFD (6.36 ± 0.76 U/mg protein) group (Figure 2A,B). In addition, hepatic and testicular CAT activity were significantly modified, similar to SOD activity (Figure 2C,D). In parallel, a significant increase in GPx activity in the liver and testis was noted in the HFD group (37.30 ± 6.69 (liver, *p* = 0.02); 14.76 ± 1.03 (testis, *p* = 0.03)) compared with the control group (43.05 ± 4.71 (liver, *p* = 0.01); 17.23 ± 2.27 (testis, *p* = 0.03)) (Figure 2E,F). In parallel, a significant decrease in GPx activity was observed in the KM and KM/HFD groups compared with the HFD group.

The results in Figure 2 show that the consumption of kefir milk (KM and KM/HFD groups) significantly corrected antioxidant enzyme activities toward normal values (*p* = 0.02) in the liver and testis organs, thus highlighting the protective effect of kefir milk against oxidative stress induced by the high-fat diet.

A strong positive correlation was observed between SOD levels in the liver and testes (r = 0.928, *p* < 0.05). Regarding CAT, a moderate but significant correlation was noted between hepatic and testicular levels (*n* = 24, r = 0.78, *p* < 0.05). In contrast, no significant correlation was observed for GPx (*n* = 24, r = 0.08, *p* > 0.05). These findings suggest that the regulation of SOD levels, and to a lesser extent CAT, is closely coordinated between the liver and testes, whereas the regulation of GPx appears to function independently within each tissue (see Supplementary Materials).

### 3.6. Effects of a High-Fat Diet and Kefir-Fermented Milk on Fatty Acid Composition and Enzyme Activity in Rat Adipose Tissue

**Figure 3:**
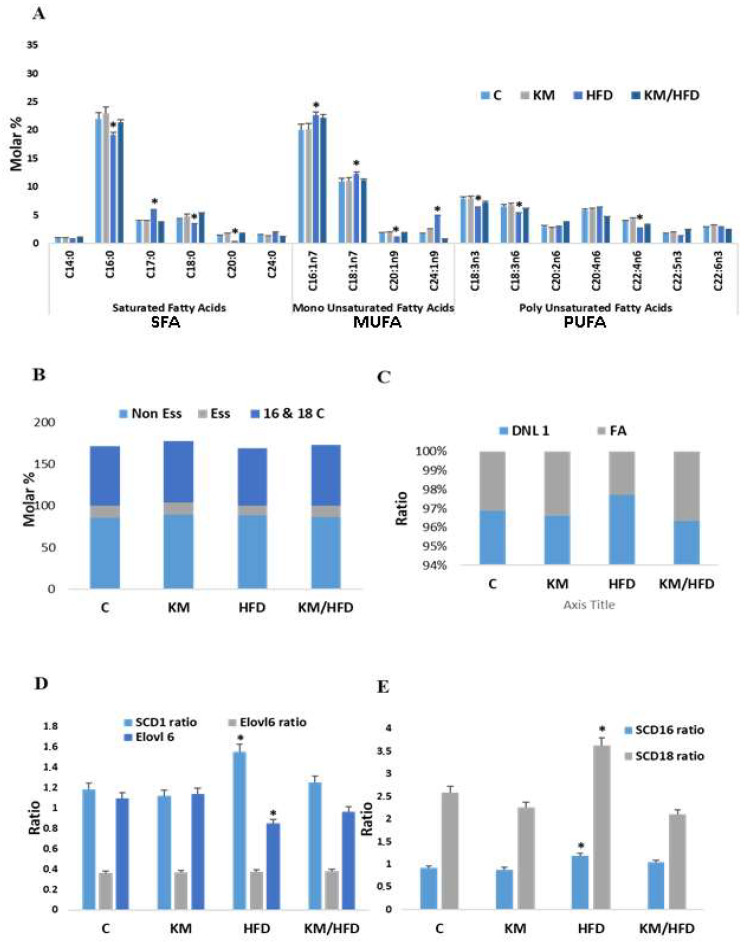

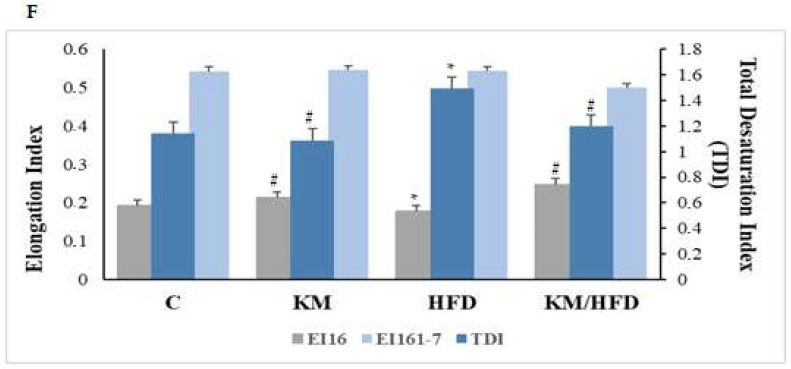
Effect of regular kefir milk consumption on fatty acid composition in adipose tissue. (**A**) Differences in the levels of saturated fatty acids (SFA), monounsaturated fatty acids (MUFA), and polyunsaturated fatty acids (PUFA) in subcutaneous adipose tissue of the control (**C**), kefir milk (KM), high-fat diet (HFD), and high-fat diet with kefir milk (HFD/KM) groups. (**B**) Composition of nonessential fatty acids (NE), essential fatty acids (**E**), and the specific fatty acids C16 and C18 in all groups. (**C**) De novo lipogenesis (DNL) index (ratio of NE to E fatty acids) and total fatty acid composition in the tested groups. A significant increase in the DNL index was observed in the HFD group compared to the control group, while kefir milk reduced the DNL index when added to the high-fat diet. (**D**) Activity ratios of Elovl6 (elongase) and SCD1 (stearoyl-CoA desaturase) in adipose tissue. These ratios reflect the relative activity of elongases and desaturases that influence fatty acid composition. The HFD group showed higher activity of these enzymes, which was partially reduced by kefir milk supplementation (HFD/KM). (**E**) Ratios of desaturase activities SCD16 (16:1/16:0) and SCD18 (18:1/18:0) in adipose tissue. These ratios are indicative of the desaturation activity of specific fatty acids and were significantly higher in the HFD group compared to controls. Kefir milk reduced these ratios, especially in the HFD/KM group. (**F**) Elongation index (EI) and total desaturation index (TDI) of white adipose tissue (WAT) in the four groups. The elongation index for 16:0 (EI16) was calculated as the ratio of 18:0 plus 18:1n-9 to 16:0, and for 16:1n-7 (EI16:1n-7), it was the ratio of 18:1n-7 to 16:1n-7. The TDI was calculated as the ratio of monounsaturated fatty acids (MUFA) (16:1n-7, 18:1n-7, 18:1n-9) to saturated fatty acids (SFA) (14:0, 16:0, 18:0). The HFD group showed an increased TDI, which was reduced by kefir milk supplementation to levels similar to the control group. The EI16 index, shown in panel F, demonstrated an increase in elongation activity in the HFD group compared to controls, with a reduction in the HFD/KM group. Abbreviations: E = essential fatty acids, NE = nonessential fatty acids, C16 and C18 = nonessential C16 and C18 fatty acids, DNL = de novo lipogenesis, FA = fatty acids, SCD = desaturation indices calculated as follows: SCD-16 = 16:1/16:0 and SCD-18 = 18:1/18:0. The asterisk (*) indicates significant differences (*p* < 0.05) compared to control rats. The hash symbol (#) indicates significant differences (*p* < 0.05) compared to HFD rats.

Figure 3A presents the fatty acid compositions in adipose tissue across four groups: control (C), kefir milk (KM), high-fat diet (HFD), and kefir milk combined with a high-fat diet (KM/HFD). The levels of nonessential fatty acids (NE), essential fatty acids (E), and specific fatty acids such as C16 and C18 showed variations depending on the dietary regimen (Figure 3B).

The NE-to-E ratio, indicative of de novo lipogenesis (DNL), was significantly elevated in the HFD group (28.23%) compared to the control group, while kefir milk notably reduced this ratio to 9.41% in the KM/HFD group (Figure 3C). The fatty acid composition was influenced by desaturases, including SCD, and the elongase Elovl6 (Figure 3C). The HFD group exhibited lower fatty acid content compared to the control and KM groups, suggesting that kefir milk helps restore the imbalance induced by a high-fat diet.

The activities of SCD and Elovl6 were analyzed as product-to-substrate ratios. The SCD desaturation indices (SCD-16: C16:1/C16:0 and SCD-18: C18:1/C18:0) increased in HFD-fed mice, reflecting a higher MUFA content in these tissues. Kefir milk supplementation reduced these indices, indicating its regulatory effect (Figure 3D). A higher Elovl6-to-SCD1 activity ratio was observed in the HFD group, followed by the KM/HFD group, corresponding with elevated NE levels (Figure 3D). Similarly, the SCD16-to-SCD18 ratio mirrored the SCD1-to-Elovl6 ratio, with kefir milk effectively lowering both in combination with a high-fat diet (Figure 3E).

The high-fat diet led to a significant rise in Elovl6 and SCD activity ratios and the DNL index (5%, 31%, and 28%, respectively) compared to controls. Kefir milk mitigated these increases, bringing them closer to control levels. In terms of elongation indices, EI16 decreased by 11% in the HFD group but was restored by kefir milk to levels comparable to the control group. The elongation index for 16:1n-7 remained unchanged (Figure 3F). Kefir milk reduced the total desaturation index by 31%, emphasizing its beneficial role in counteracting the metabolic disturbances induced by a high-fat diet.

### 3.7. Histology Study

#### 3.7.1. Structural Observation of Kefir Grains

**Figure 4:**
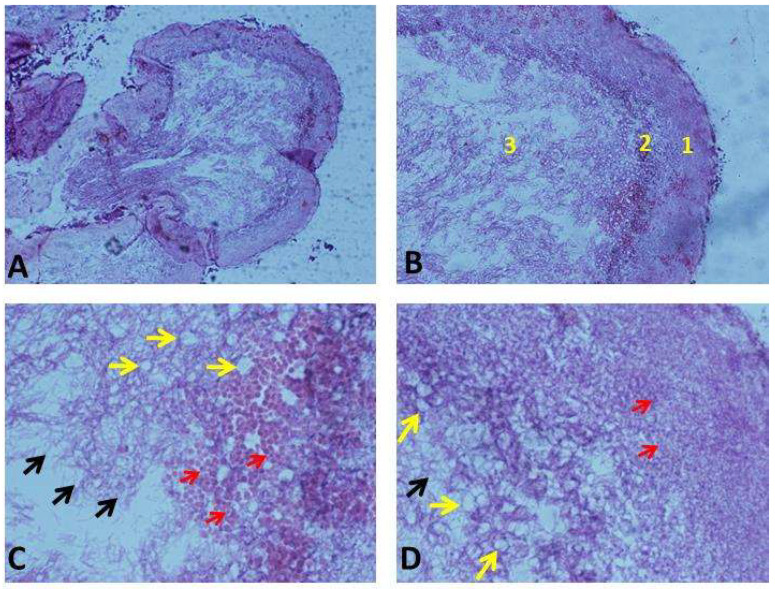
Histological sections of kefir grains. (**A**) General shape of the kefir grains (4×); (**B**) histological sections showing three portions that form the kefir grains: (1) outer or peripheral area, (2) submiddle or dense intermediate area, and (3) inner or central filamentous area (10×); (**C**,**D**) histological sections revealing the presence of fibrillian material and different forms of bacteria and yeasts: yellow arrows show spores, red arrows show round bacteria, and black arrows show rod-shaped bacteria (40×).

For the first time, histological sections of the kefir grains were evaluated (Figure 4). The observations of the Kefir sections revealed three areas: the outer, submiddle, and inner portions (Figure 4A,B). Additionally, histological sections revealed the presence of different forms of bacteria and yeasts (Figure 4C,D). Lactobacilli, yeast, and fibrillar material were observed. The fibrillar material was most likely the polysaccharide kefiran. Three different types of lactobacilli (short, long, and curved) were observed embedded in the grain along with yeast cells. Kefir grains had a spongy, fibrillar structure that was branched and interconnected. The amount of fibrillar material increased progressively toward the interior portions of the grains.

#### 3.7.2. Observation of the Tissue Structure of Internal Organs

Multiple fields from at least three sections of each tissue (liver, pancreas, and heart) were evaluated to ensure the reliability of our results.

**Figure 5:**
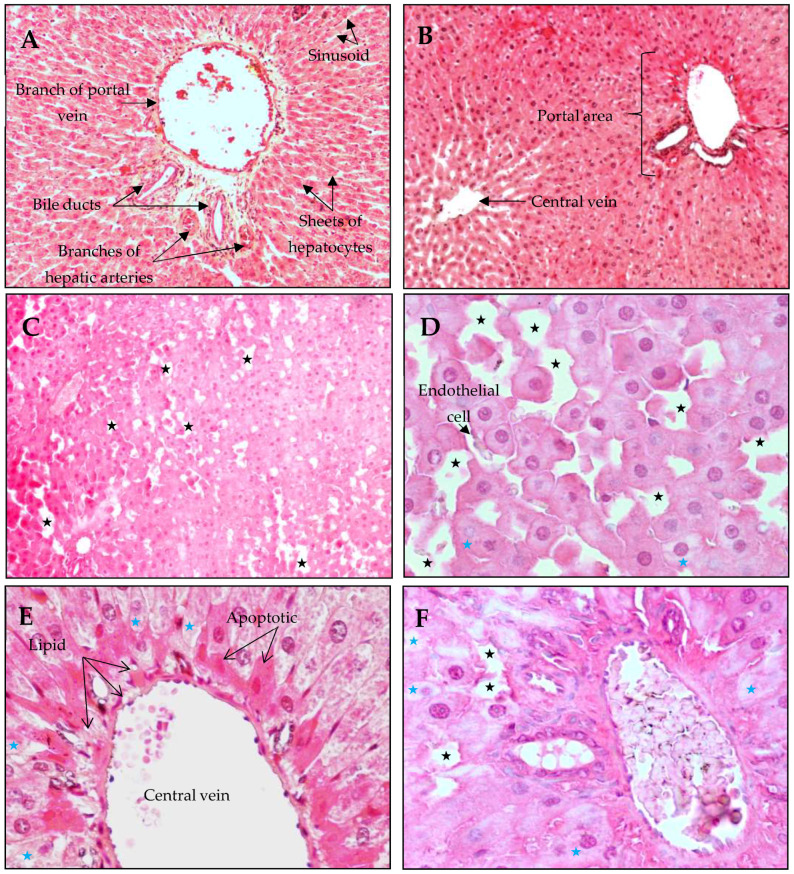
Microscopic observation of the livers of the rats (H&E). (**A**) Liver sections of a control rat (**C**) showing a normal structure of hepatic tissue (10×). (**B**) Normal architecture of hepatic tissue from the kefir milk group (KM) (10×). (**C**–**E**) Changes in the liver structure of HFD-fed rats showing important dilated sinusoids (black stars) altered by hepatocytes (blue stars), the presence of some apoptotic cells located in the central vein, and an abundance of lipid droplets and Küppfer cells (10× and 40×). (**F**) Microscopic observation of the liver in the KM/HFD group showing a normal portal vein with the presence of some dilated sinusoids (40×).

Compared with those of control rats, histological examination revealed numerous changes in the liver architecture of treated rats (Figure 5). Indeed, a normal aspect of liver tissue was observed in rats treated with kefir milk (KM) compared with control rats (C), with prominent structures of the liver, such as the portal area and the centrolobular vein (Figure 5A,C). In contrast, several structural alterations were detected in the hepatic tissue of HFD-fed rats, such as important dilation of the sinusoids (Figure 5C–E) and the presence of foamy hepatocytes with no homogenous staining (pale cytoplasm), which reflects a certain lack of organelles (Figure 5E). The foamy appearance was probably due to the dilatation of the hepatocyte endoplasmic reticulum. Additionally, some apoptotic hepatocytes were located mainly near the central vein, with an important accumulation of lipid droplets (Figure 5E). These disturbances were reduced in the KM/HFD-fed liver in the presence of some dilated sinusoids and a few altered hepatocytes (Figure 5F).

**Figure 6:**
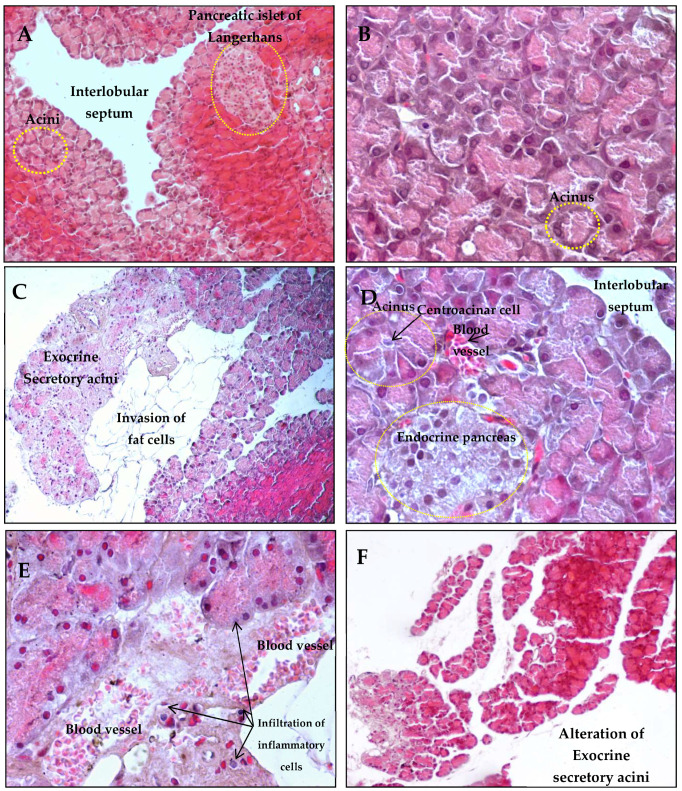
Hematoxylin—eosin (H&E)-stained pancreas sections from the rats. (**A**) Pancreatic representative image of a control rat (**C**) showing a normal structure of exocrine (acini) and endocrine (islets of Langerhans) pancreas (10×). (**B**) No structural changes were detected in the pancreatic tissue of KM rats (40×). Invasion of fat cells (**C**) (10×), blood vessel dilatation, and inflammatory cell infiltration in pancreatic tissue (**D**,**E**) were detected in pancreas sections of rats that consumed a high-fat diet (HFD) (40×). (**F**) Alteration of exocrine secretory acini in the KM/HFD group.

Microscopic observation of the pancreatic tissue of control rats (Figure 6A) revealed a normal architecture in the KM group (Figure 6B). On the other hand, some structural modifications, such as the invasion of adipose cells (Figure 6C) and dilated vessels that are full of red blood cells reflecting a certain degree of fatigue in this group (Figure 6D,E), were observed in HFD-fed rats. The infiltration of some inflammatory cells was noted in HFD-fed rats (Figure 6E). In parallel, an alteration in the number of exocrine secretory acini was detected in the pancreatic tissue of the KM/HFD group (Figure 6F). These observations show that the consumption of kefir has no significant effect on the pancreas of rats fed a high-fat diet.

**Figure 7:**
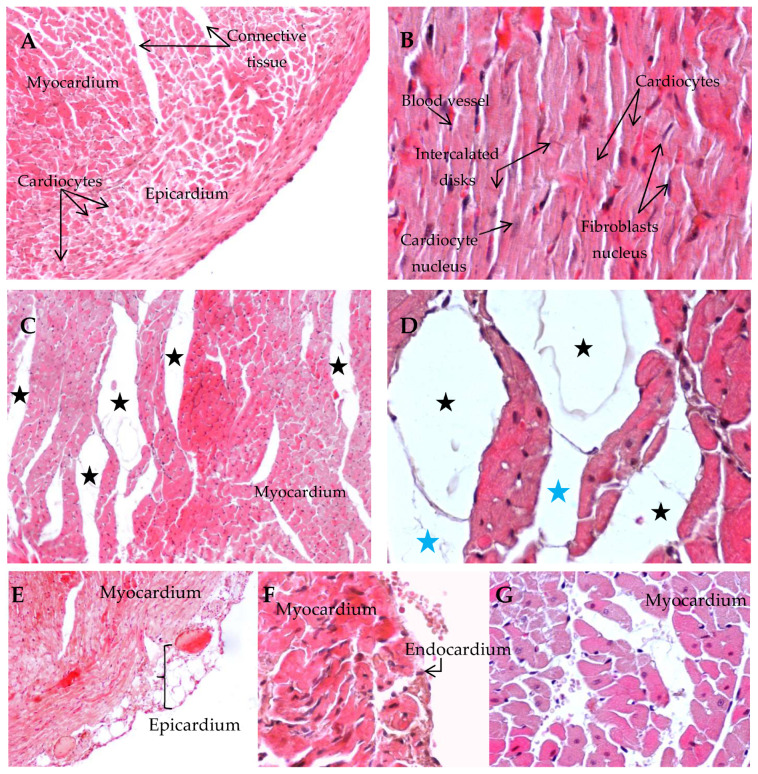
Microscopic observation of cardiac tissue (H&E). (**A**) Normal structure of the myocardium in control rats (10×). (**B**) Cardiac tissue of the KM group showing normal cardiomyocytes with central nuclei and intracellular contractile myofilaments and intercalated disks between cells and fibroblasts located in connective tissue (40×). (**C**,**D**) Dilation of veins, represented by black stars (10×), and accumulation of adipose cells, represented by blue stars (40×), were noted in the myocardium of the HFD group. (**E**–**G**) A normal architecture of cardiac tissue was observed in different histological sections of the hearts of KM/HFD-fed rats (40×).

A histological study revealed normal myocardial architecture in the control group (Figure 7A). The same phenomenon was observed in the cardiac tissue of the KM group, with branching and anastomosing striated cardiocytes with a central nucleus and intracellular contractile myofilaments. Intercalated disks join individual cardiocytes (Figure 7B). The myocardium of the HFD group showed important dilated veins (Figure 7C) and the accumulation of adipose cells in cardiac tissue (Figure 7D). However, the cardiac tissue in the KM/HFD group presented a normal myocardium and epicardium architecture (Figure 7E) with intact blood vessels and cardiocytes (Figure 7F,G). In general, no significant alterations were noted in the hearts of the rats treated with kefir or a high-fat diet.

### 3.8. Molecular Docking Studies

**Figure 8:**
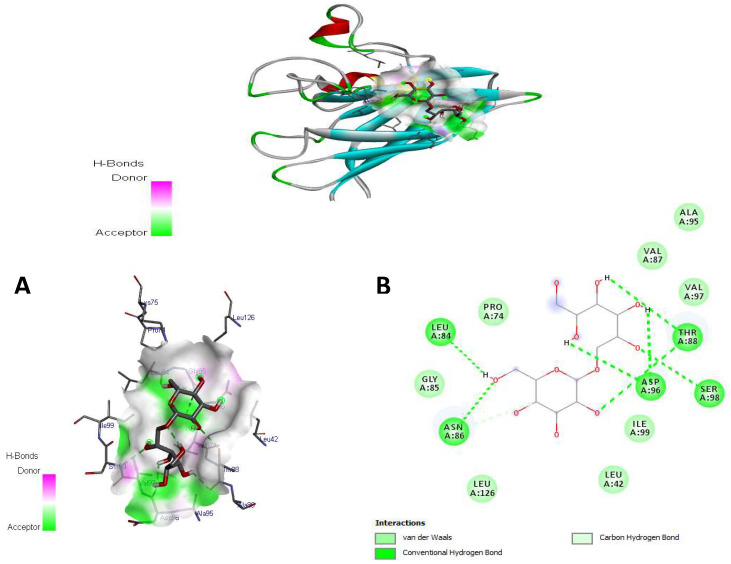
3D view (**A**) and 2D (**B**) view of the complex docking obtained between the docked conformation of kefiran (1) in the binding receptor of SOD (PDB: 4 mcm).

The docking simulations were run on the major compounds of kefir milk to identify the active site of amino acid residues involved in protein–ligand interactions. Kefiran showed various values of binding energy against SOD (4 mcm), CAT (1 dgb), GPx (3 kij), and NADPH oxidase (Table 5). Kefiran demonstrated suitable interactions with several amino acid residues for SOD in 2D, as illustrated in Figure 8, with a binding energy of -5.1 kcal/mol. Indeed, conventional hydrogen bonds are known as the most important factor in proving the stability of the docking complex and, by extension, the ligand’s inhibitory activity on the receptor.

Numerous intermolecular interactions are shared between our molecules, including seven standard hydrogen bonds of the oxygen (OH group) LEU84, ASN86, THR88, ASP96, and SER98 and seven van der Waals forces with the PRO74, LEU 42, 126, GLY85, ILE99, VAL 97, 87, and ALA95 amino acid residues. In addition, one carbon—hydrogen bond with ASN86.

**Figure 9:**
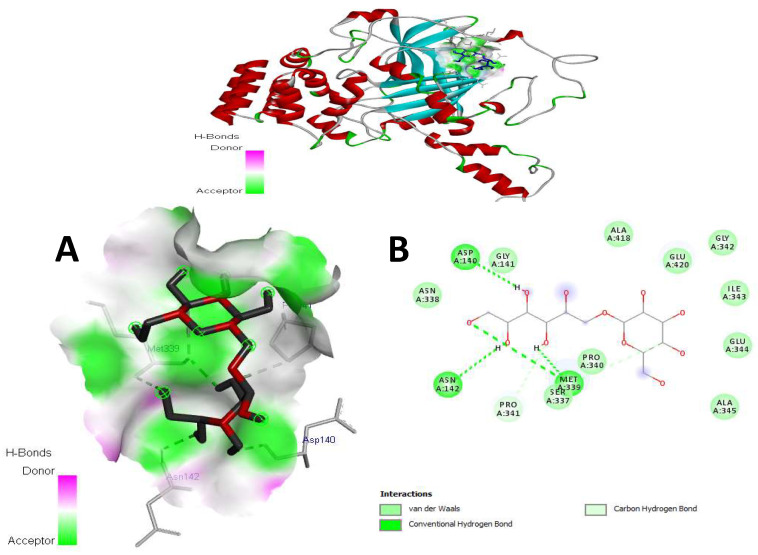
3D view (**A**) and 2D (**B**) view of the docked kefiran molecule (1) within the binding targets of CAT (PDB: 1 dgb).

Figure 9 shows the different interactions of kefiran with its target receptor. The related binding energy is −5.4 kcal/mol, as presented in Table 5. Four conventional hydrogen bonds are created between the ligand OH groups, such as ASP 140, ASN 142, and MET 339. Additionally, we obtained numerous van der Waals interactions with the amino acid residues GLY 141, 338, PRO 340, SER 337, ALA 345, GLU 344, ILE 343, GLY 342, GLU 420, and ALA 418. We also observed a carbon—hydrogen bond interaction with PRO341.

**Figure 10:**
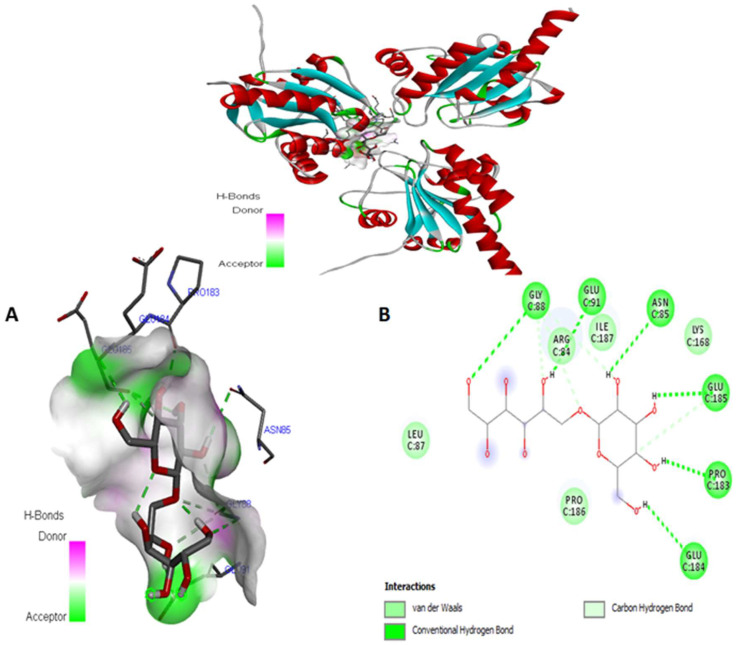
3D view (**A**) and 2D (**B**) binding modes of the interactions between the docked conformation of kefiran (1) and the binding site of GPx (PDB: 3 kij).

Additionally, the third antioxidant activity was evaluated by the target protein GPx, encoded by 3kij.pdb, which is associated with kefiran. The binding energy was −5.3 kcal/mol. Six conventional hydrogen bonds were found with GLY88, PRO 186, GLU 91, ASN 85, GLU 185, 184, and PRO183. Five van der Waals bonds interact with the amino acids LEU 87, ARG84, ILE187, LYS168, and PRO186. Additionally, four carbon—hydrogen bonds were observed between our ligand and ARG 84, GLY 88, and GLU 185 amino acid residues of the target receptor in chain A, as revealed in Figure 10.

Finally, the most important binding energy was −6.4 kcal/mol, which was associated with the kefiran-NADPH oxidase interaction. Ten H-bonds were shown with THR9, THR112, MET33, TYR31, HIS79, VAL81, ASN248, and ALA11.

**Figure 11:**
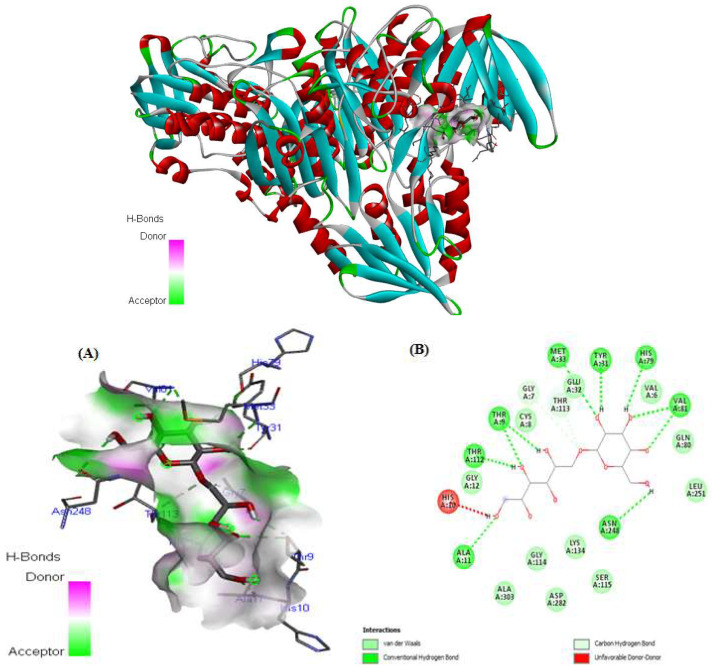
Molecular docking of kefiran (**1**) (gray and red) and its interactions (**A**) and (**B**) with the active site of the crystal structure of NADPH oxidase (PDB code: 2CDU).

Nine van der Waals bonds were formed with the amino acids ALA303, ASP282, GLY114, VAL6, GL80, LEU251, LYS134, SER115, CYS8, GLY12, and GLU32. In addition, two carbon—hydrogen bonds were formed between our molecule and the GLY7 and THR113 residues of the site receptor, as shown in Figure 11. These interaction connections between our molecules and the protein are implicated in complex stabilization and contribute to its inhibitory and antioxidant properties.

## 4. Discussion

The present study provides valuable insights into the potential benefits of kefir milk, particularly in the context of obesity and related metabolic disorders. One of the key strengths of our work is the integration of a multidisciplinary approach, combining biochemical, histopathological, and in silico analyses to explore the effects of kefir. Notably, we highlight the use of in silico molecular modeling to study the interactions of kefiran with antioxidant enzymes, an approach rarely applied in kefir research. Moreover, our study explores the combined effect of kefir and a high-fat diet, an area that has not been sufficiently examined in the literature. By investigating the bioactive polysaccharide kefiran, we have identified potential molecular mechanisms that may explain the metabolic health benefits of kefir. These findings open new avenues for using kefir as a natural therapeutic agent in the management of obesity and associated diseases.

Our study highlights that kefir milk exhibits a significantly higher total antioxidant activity compared to both commercial milk and kefir grains. This enhanced antioxidant capacity of kefir, which is a result of the fermentation process, aligns with previous studies, such as that of Grishina et al. (2011) [33], who found that the antioxidant capacity of kefir samples was roughly three times greater than that of regular milk. This increase is likely due to the proteolytic activity of the microflora during fermentation, which enhances the bioactive properties of kefir, as noted in various studies. Collectively, these findings emphasize the potential health benefits of kefir, especially in relation to metabolic disorders and obesity.

As previously indicated, the KM and KM/HFD groups showed a notable reduction in body weight compared to the HFD group of rats [16]. This weight loss was associated with a significant decrease in fat mass, implying that regular intake of kefir milk may help prevent weight gain and fat accumulation induced by a high-fat diet. Our findings align with earlier research that explored how kefir acts to mitigate fat mass increase by inhibiting lipogenesis and modifying oxidative stress markers [34,35]. However, our results differ from other studies that observed weight gain following oral kefir consumption [36,37]. Additionally, we did not find any significant changes in liver weight among the various groups compared to the HFD group. This variation could be attributed to differences in dosage (1 mL versus 200 µL) and the duration of treatment (8 weeks versus 2 weeks). Some studies suggest that the weight gain may be linked to kefir’s role in enhancing the gastrointestinal microbiota, which aids in nutrient digestion and provides additional vitamins, enzymes, and amino acids to the rats.

Several studies have highlighted the direct link between overweight or obesity and lipid and carbohydrate profiles in human and animal models [38,39]. Obesity is invariably associated with increases in plasma triglycerides, total cholesterol, and LDL levels [40]. Lipids are critical substances involved in a wide range of cellular processes and homeostasis. The liver is crucial for lipid metabolism, acting as the primary site for the uptake, synthesis, and release of lipoproteins into the bloodstream. Obesity disrupts lipid levels in the body, and these disturbances can signal liver and cardiovascular issues. An abnormal lipid profile is a significant marker of liver dysfunction [6,40]. In our study, we observed elevated levels of total cholesterol (TC) and triglycerides (TG) in the livers of rats that were given a high-fat diet (HFD). These results are consistent with our earlier research, which indicated that obesity negatively impacts plasma lipid levels, leading to increased triglycerides, cholesterol, and LDL, while decreasing HDL levels [16,39].metabolism in obese rats. Numerous studies suggest that lactic acid bacteria such as Lactobacillus plantarum, kefiran, and vitamins D and B5 have lipid-lowering effects [36,41,42], but their mode of action has not been adequately identified [43,44]. These components of kefir have the potential to inhibit fat synthesis and storage, as well as the expression of genes such as adenosine monophosphate-activated protein kinase-α (AMPK-α), fatty acid synthase (FAS), acetyl-CoA carboxylase (ACC), and peroxisome proliferator-activated receptor γ (PPAR-γ). Additionally, they promote glucose uptake by enhancing the expression of genes related to hepatic lipid metabolism, including lipoprotein lipase (LPL), insulin receptor substrate 2 (IRS2), protein kinase Bβ (Akt2), and AMPK in preadipocytes. Furthermore, there is a reduction in the absorption of dietary cholesterol in the intestines or a disruption in cholesterol synthesis, leading to decreased serum levels of total and LDL cholesterol and triglycerides, while HDL cholesterol levels increase [44,45].

Hepatic glucose levels were also evaluated. The liver is crucial for maintaining normal glucose levels throughout the body by regulating de novo glucose production (gluconeogenesis) and glycogen breakdown (glycogenolysis), thereby controlling the release of glucose from the liver. In our previous research, we found no significant differences in plasma glucose levels among the groups or within them [16]. The same pattern was observed in the liver in the current study. This finding contrasts with the research conducted by Ostadrahimi et al. (2015) [46], which indicated that kefir could serve as a beneficial complementary or adjunct therapy for diabetes, as it lowered glycemia and HbA1c levels in patients with type 2 diabetes without affecting the serum lipid profile [46]. The discrepancy in results may be attributed to differences in dosage (600 mL/day/patient compared to 1 mL/rat) and treatment duration (8 weeks versus 6 weeks).

The liver serves as the primary organ in the body for detoxification and protein synthesis, with various enzymes playing a key role in these functions. The concentrations of enzymes and proteins released by the liver into the bloodstream can be utilized to evaluate liver function. Commonly measured serum levels include alanine aminotransferase (ALT), aspartate aminotransferase (AST), lactate dehydrogenase (LDH), and bilirubin, which are all important indicators of liver health [47].

Obesity has been associated with the levels of serum liver enzymes [47]. In this study, the results revealed elevated levels of AST, ALT, and LDH in HFD-fed rats. However, kefir milk consumption resulted in relatively lower enzyme levels. Our results are consistent with recent studies showing an association between obesity and the serum levels of transaminases [48,49]. In the same context, the bilirubin level was evaluated to assess liver function [6,49,50]. In our study, no change in this marker was observed after treatment with kefir milk and/or a high-fat diet. Our observation was not similar to that of a previous study showing an increase in bilirubin levels after consumption of a high-fat diet [49]. The hepatoprotective capacity of kefir was also assessed by Golli-Bennour et al. (2019) [51], who investigated the effects of kefir on pesticide-induced hepatotoxicity.

Creatinine levels were measured to assess renal function. Body mass index (BMI) is positively associated with the risk of developing end-stage renal diseases [52]. In addition, overweight and obesity are important risk factors for chronic kidney disease because of their close associations with diabetes and hypertension [53]. An HFD results in increased creatinine levels, which are important indicators of kidney dysfunction.

Consistent with its in vitro antioxidant and radical scavenging activity, kefir milk was also shown to possess potent antioxidant properties in vivo. Our results demonstrate the significant impact of kefir milk (KM) on mitigating oxidative stress induced by a high-fat diet (HFD), with a particular focus on liver and testis tissues. The reduction in lipid peroxidation (MDA levels) and the enhancement of antioxidant enzyme activities (SOD, CAT, GPx) highlight the systemic antioxidative effects of KM. Strong correlations observed in oxidative stress markers, such as MDA and thiols, between the liver and testes suggest coordinated regulatory mechanisms across these tissues. However, the lack of correlation for GPx activity between the two tissues indicates a tissue-specific regulation, emphasizing the localized roles of certain antioxidants in addressing oxidative challenges. These findings reinforce the role of kefir milk as a potent modulator of oxidative stress, providing systemic protection while allowing tissue-specific adaptations to oxidative damage. By restoring the balance between pro-oxidants and antioxidants, KM demonstrates its potential to attenuate oxidative stress and protect against HFD-induced metabolic and organ damage. These results support the inclusion of KM as a functional food to combat oxidative stress-related pathologies associated with obesity.

This antioxidant activity of kefir milk may be due to the relatively high levels of total phenolics and flavonoids found in this beverage. Polyphenolic compounds can inhibit the enzyme xanthine oxidase, which plays a role in generating superoxide radicals and stimulating the activity of antioxidant enzymes [54]. These phenolic compounds can function as antioxidants in two ways: directly, by interacting with both radical and non-radical reactive oxygen species (ROS), and indirectly, by influencing the gene expression of antioxidant proteins and enzymes [55]. Our hypothesis is supported by other research highlighting the antioxidant properties of kefir, such as the study conducted by Güven et al. (2003) [56]. In their toxicity test involving carbon tetrachloride (CCl4) in mice, kefir demonstrated a stronger antioxidant effect compared to vitamin E. Furthermore, Ozcan et al. (2009) [57] investigated the impact of kefir supplementation in rats subjected to oxidative stress induced by lead (Pb). After 6 weeks of treatment, the consumption of kefir led to an increase in glutathione peroxidase activity and a reduction in malondialdehyde levels, bringing them down to levels similar to those of the non-induced group. These findings reinforce the hypothesis that kefir may serve as a valuable tool for managing oxidative stress.

The liver is crucial for lipid metabolism. As a result, lipids, primarily triglycerides, can accumulate in the liver, particularly in hepatocytes, when there is an imbalance between the intake of fat from the diet or adipose tissue and the export of fat as part of very-low-density lipoproteins [58]. Typically, small lipid droplets can only be seen using electron microscopy or fat staining techniques and are found in the cytoplasm of hepatocytes [59]. However, in situations of metabolic imbalance, stress, or cellular damage associated with various pathological conditions, these lipid droplets can grow large enough to be observed with light microscopy as distinct vacuoles within the hepatocyte cytoplasm [60]. In the present study, histological observations of different tissues revealed several structural changes induced by a high-fat diet. Compared with those in the other groups, the hematoxylin—eosin-stained sections revealed an accumulation of adipocytes in HFD-fed rats. The increase in lipid droplets was caused by a high-fat diet. Thus, the increase in lipids in the blood triggered an increase in fat accumulation in the liver, pancreas, and heart tissues.

Adipose tissue is a dynamic and metabolically active organ that plays a critical role in energy homeostasis and endocrine function. Triacylglycerols are the major lipid storage form of fatty acids in white adipose tissue. Triacylglycerols contain a complex mixture of fatty acids whose molecular structures differ greatly. In fact, adipocyte triacylglycerols contain a wide range of fatty acids, ranging in chain length from 12 to 24 carbon atoms and in unsaturation from 0 to 6 double bonds [61]. The nature of dietary fats could influence lipid metabolism and body fat accumulation [62]. High-fat consumption is closely associated with the development of nutritional obesity. Indeed, the type of dietary fat and the fatty acid composition may influence lipid metabolism and body fat deposition. Our results are consistent with several studies reporting that the consumption of a diet high in (saturated) fat stimulates fat storage in the abdominal or visceral regions [63,64].

Over 85% of the fatty acids present in the adipose tissue triglycerides of the four groups of rats were nonessential (NE) fatty acids. This percentage is consistent with other findings [65,66]. Previous studies have examined the fatty acid composition of adipose tissue in control mice and mice fed a high-fat diet, but they did not control for the effect of dietary fatty acid composition. This important confounding factor makes it difficult to interpret conclusions regarding the effect of a high-fat diet (HFD) on the activity of the two enzymes Elovl6 and SCD1 [66].

Desaturases are enzymes that introduce double bonds into fatty acids. Among these, stearoyl-CoA desaturase (SCD) is the most significant, serving as a crucial regulator of de novo lipogenesis [67]. This enzyme catalyzes the production of the monounsaturated fatty acids (MUFA) palmitoleic acid (C16:1n7) and oleic acid (C18:1n9) from their corresponding saturated fatty acids (SFA), palmitic acid (C16:0) and stearic acid (C18:0), respectively. MUFA are essential for synthesizing various lipids, including triglycerides, cholesterol esters, and phospholipids, which are vital for forming cell membranes and functioning as lipid signaling molecules [67]. Our results suggest an important role for SCD1 in de novo lipogenesis. The overexpression of SCD1 in mouse subcutaneous white adipose tissue (scWAT) indicates that SCD1 can induce lipolysis by regulating lipase and lipophagy pathways, thereby promoting fat mobilization and energy expenditure.

Elovl6 exhibits a higher specificity for palmitate compared to palmitoleate. The position of the C18:1 double bond is crucial for differentiating between fatty acids derived from palmitate elongation (C18:0 and C18:1n9) and those from palmitoleate elongation (C18:1n7). We calculated ratios for palmitate elongation (EI16) [(C18:0 + C18:1n9)/C16:0] and palmitoleate elongation (EI16:1n7) [C18:1n7/C16:1n7]. By dividing the palmitate elongation equation by the palmitoleate elongation equation, we determined that palmitate is the preferred substrate for Elovl6 [65]. In our findings, Elovl6 demonstrated the same preference for both palmitate and palmitoleate in both mice and humans [66].

Adipocytes are distinguished by their large lipid droplets rich in triglycerides (TAGs), which are encased in a monolayer of phospholipids. TAGs serve as a biological fuel source and possess twice the caloric density of an equivalent mass of protein or carbohydrates. Obesity arises from a chronic positive energy imbalance and is marked by the accumulation of TAG-rich adipocytes [66,68]. TAGs are neutral lipids made up of a glycerol backbone esterified with three fatty acids. Notably, the relative increase in desaturation between adipose tissue and the consumed diets remained consistent; mice on a standard diet exhibited a lipid composition in their adipose tissue that was comparable to that of those on a high-fat diet. Regarding specific fatty acids, the rise in fatty acid chain length and desaturation rates was primarily attributed to an increase in oleate proportion and a decrease in palmitate proportion [66].

Our experimental results suggest that kefir milk has significant antioxidant potential. To explore this hypothesis, we performed accurate structural predictions of key antioxidant enzymes, including superoxide dismutase (SOD; PDB: 4 mcm), catalase (CAT; PDB: 1 dgb), and glutathione peroxidase (GPx; PDB: 3 kij). Kefiran was selected as a candidate ligand for molecular docking simulations due to its promising bioactive properties. These enzymes were chosen because of their critical role in the antioxidant defense system, which is essential for protecting cells from oxidative stress and damage [68].

NADPH oxidases, a family of membrane-bound electron transport proteins, play a key role in initiating the generation of reactive oxygen species (ROS). By transferring electrons to molecular oxygen (O_2_), these enzymes generate superoxide anions (O_2_●−), which contribute to the formation of various ROS [69,70].

Figure 8, Figure 9, Figure 10 and Figure 11 show that the docked ligand fits well into the binding cavities of the target proteins. Variations in the binding interactions and energies of kefiran with the antioxidant enzymes were observed, likely due to differences in binding site occupancy, as detailed in Table 5 and Figure 8, Figure 9, Figure 10 and Figure 11. Docking simulations revealed that specific amino acid residues in SOD, CAT, GPx, and NADPH oxidase were involved in hydrogen bond formation, demonstrating the high conformational stability of these complexes.

Our in silico results are consistent with the in vivo results, suggesting that the enhanced interactions of kefiran with antioxidant enzymes contribute to the increased activity of SOD, CAT, and GPx in obese rats consuming kefir milk (KM/HFD) compared to HDF rats (in vivo, as shown in Figure 2), alongside the inhibition of NADPH oxidase activity (in silico). Kefiran showed robust docking results with binding affinities of −6.4 kcal/mol for NADPH oxidase, −5.1 kcal/mol for SOD, −5.4 kcal/mol for CAT, and −5.3 kcal/mol for GPx. These stable interactions were reinforced by the formation of numerous van der Waals forces and other non-covalent contacts. Comparable in silico studies reported binding affinities for SOD (−4.5 to −8.7 kcal/mol), CAT (−6.6 to −9.18 kcal/mol), GPx (−5.6 to −8.22 kcal/mol), and NADPH oxidase (−6.27 to −10.62 kcal/mol) [71,72,73]. This pattern supports the potential of kefiran as a potent antioxidant.

Interestingly, the reference antioxidant L-ascorbate, known for its high potency, showed similar binding energies (−5.3, −6.6, and −5.6 kcal/mol, respectively) with SOD (4 mcm), CAT (1 dgb), and GPx (3 kij). It also showed specific interactions with key amino acid residues [71,74]. Furthermore, kefiran exhibited potent inhibition of NADPH oxidase with a higher binding energy (−6.4 kcal/mol) and more conventional hydrogen bonds compared to the other enzymes.

These docking studies highlight the robust binding affinity of kefiran for SOD, CAT, GPx, and NADPH oxidase, with binding patterns very similar to those of L-ascorbate. These results underline the potential of kefiran as a highly potent antioxidant capable of interacting with multiple enzymes in the antioxidant defense system.

## 5. Conclusions

In conclusion, chronic consumption of kefir milk at a dose of 1 mL/100 g body weight appears to effectively control obesity and its associated metabolic disorders. Our previous studies have demonstrated its beneficial effects on spermatogenesis impairment in obese rats, further highlighting its potential as a therapeutic agent. The observed improvements in serum and liver lipid profiles, as well as oxidative stress markers, underscore the protective role of kefir milk in combating obesity-related complications. Additionally, these positive effects extend to other organs, including the testes, pancreas, and heart, indicating a broader physiological impact. Importantly, enhancing redox balance pathways through kefir milk supplementation represents a promising strategy to mitigate the adverse consequences of obesity. For the first time, our in silico findings reveal that kefiran, the key bioactive polysaccharide in kefir, can effectively increase the activity of antioxidant enzymes, including superoxide dismutase (SOD), catalase (CAT), and glutathione peroxidase (GPx), while simultaneously reducing NADPH oxidase activity. These results are consistent with our in vivo observations, confirming kefiran’s strong antioxidant properties. Our molecular docking analysis further supports these findings, providing valuable insights into the mechanisms through which kefiran exerts its antioxidant effects. This study paves the way for future in silico investigations aimed at exploring the antioxidant potential of other kefir bioactive compounds. Based on our experimental and computational results, we conclude that kefir milk has significant potential as a natural source of antioxidant agents. These findings suggest promising applications for kefir-derived products in the pharmaceutical industry, particularly in the development of functional foods or nutraceuticals aimed at mitigating oxidative stress and related metabolic disorders. This study presents interesting results, but several limitations should be considered. First, the animal model used (rats) may not fully reflect the effects in humans. Additionally, the study duration (60 days) and sample size (30 rats) may limit the generalizability of the results. Lastly, the variability in the kefir used, depending on the preparation method, may also influence the observed effects. Further research is needed to confirm these results and evaluate their impact on humans.

## Figures and Tables

**Table 1:** Total antioxidant capacity (TAC) and total phenolic content (TPC) of commercial milk and kefir milk.

	Proteins (mg/mL)	TPC (mg GAE/100 mL of Milk)	TAC Methods		
			DPPH (mg/100 mL)	ABTS (mg/100 mL)	FRAP (mg/100 mL)
Commercial milk	30.23 ± 1.30	65 ± 1.35	3.2 ± 0.05	15 ± 0.36	1.29 ± 0.02
Kefir milk	28.04 ± 1.98	97.05 ± 1.06	4.2 ± 0.03	25 ± 0.42	0.24 ± 0.01

Data are presented as the mean ± SD, GAE: gallic acid equivalent, DPPH = 2,2-diphenyl-1-picrylhydrazyl assay; ABTS = 2,2-azinobis (3-ethylbenzothiazoline-6-sulfonic acid) assay; FRAP = ferric reducing antioxidant power assay.

**Table 2:** Chronic effect of kefir milk consumption changes in body weight (bw) as well as fat and liver relative weights.

	C	KM	HFD	KM/HFD
Initial body weight (g)	188.33 ± 7.63	183 ± 5.16	186.66 ± 10.99	186.66 ± 4.08
Final body weight (g)	288.33 ± 6.83	265 ± 7.74 *^,#^	406.70 ± 17.43 *	318 ± 24 ^#^
Fat weight (g/100 g bw)	2.58 ± 0.50	2.29 ± 0.26 ^#^	4.85 ± 0.47 **	2.82 ± 0.45 ^#^
Liver relative weight (g/100 g bw)	3.72 ± 0.43	3.67 ± 0.31	3.93 ± 0.39	3.52 ± 0.24

Animals were treated with kefir milk at a 1 mL/100 g bw dose for 60 days. (C): control rat consumed a normal diet; (KM): normal diet + 1 mL/100 g bw of kefir milk; (HFD): high-fat diet; and (KM/HFD): high-fat diet + 1 mL/100 g bw of kefir milk. Data are expressed as mean values (*n* = 6). The asterisk (*) indicates significant differences (*p* < 0.05) or (**) (*p* < 0.01) compared to control rats. The hash symbol (^#^) indicates significant differences (*p* < 0.05) compared to HFD rats.

**Table 3:** Chronic effect of kefir milk consumption (1 mL/100 g bw) on liver metabolic parameters.

Groups	Total Cholesterol (TC) (g/L)	Triglycerides (TG) (g/L)	Glucose (g/L)	Total Proteins (g/L)
C	7.30 ± 0.71	9.50 ± 0.49	1.64 ± 0.69	49.21 ± 6.11
KM	6.28 ± 0.68 *^,#^	7.44 ± 0.66 *^,#^	1.84 ± 0.52	48.83 ± 5.52 ^#^
HFD	9.22 ± 0.49 **	11.05 ± 0.46 **	1.79 ± 0.84	36.27 ± 3.64 **
KM/HFD	7.15 ± 0.47 ^#^	9.07 ± 0.51 ^#^	1.49 ± 0.81	42.16 ± 2.59 ^#^

The effect of kefir milk consumption on metabolic parameters in the liver. Animals were treated with kefir milk at a 1 mL/100 g bw dose for 60 days. (C): control rat consumed a normal diet; (KM): a normal diet + 1 mL/100 g bw of kefir milk; (HFD): high-fat diet; and (KM/HFD): high-fat diet + 1 mL/100 g bw of kefir milk. Data are expressed as mean values (*n* = 6). The asterisk (*) indicates significant differences (*p* < 0.05) or (**) (*p* < 0.01) compared to control rats. The hash symbol (^#^) indicates significant differences (*p* < 0.05) compared to HFD rats.

**Table 4:** Chronic effect of kefir milk on liver and kidney functions.

Groups	ALT (UI/L)	AST (UI/L)	LDH (UI/L)	Bilirubin Total (µmol/L)	Creatinine (µmol/L)
C	97.09 ± 8.67	146.70 ± 25.74	489.66 ± 105.32	2.06 ± 0.41	60.00 ± 8.94
KM	91.54 ± 10.09 ^#^	149.95 ± 12.48 ^#^	482 ± 69.57 ^#^	1.92 ± 0.33	61.66 ± 12.50
HFD	119.66 ± 10.66 *	182.74 ± 19.36 *	661.33 ± 105.57 *	2.15 ± 0.58	73.33 ± 10.60 *
KM/HFD	98.37 ± 9.58 ^#^	147.46 ± 34.19 ^#^	471.50 ± 79.15 ^#^	2.07 ± 0.36	65 ± 16.43

Chronic effect of kefir milk consumption (1 mL/100 g bw) on serum parameters highlighting liver and kidney functions. (C): control rat consumed a normal diet; (KM): normal diet + 1 mL/100 g bw of kefir milk; (HFD): high-fat diet; and (KM/HFD): high-fat diet + 1 mL/100 g bw of kefir milk. Data are expressed as mean values (*n* = 6). The asterisk (*) indicates significant differences (*p* < 0.05) compared to control rats. The hash symbol (^#^) indicates significant differences (*p* < 0.05) compared to HFD rats.

**Table 5:** Molecular docking analysis of kefiran with diverse antioxidant enzymes.

		Intermolecular Interractions	
Protein Complexes	Docking Energy(kcal/mol)	Conventional Hydrogen Bonds	Interacting Amino Acid Residues
**SOD**	−5.1	7	PRO74, LEU84, 42, 126, GLY85, ASN 86, ILE99, ASP96, THR88, VAL97, 87, ALA95
**CAT**	−5.4	4	ASP140, GLY141, ASN142, 338, PRO341, 340, SER337, MET339, ALA345, GLU344, ILE343, GLY342, GLU420, ALA418
**GPX**	−5.3	6	GLY88, LEU87, PRO186, ARG84, GLU91, ILE187, ASN85, LYS168, GLU185, 184, PRO183, 186
**NADPH oxidase**	−6.4	10	THR9, CYS8, GLY7, 12, TYR31, MET33, GLU32, THR112, 113, HIS79, VAL6,81, GLN80, LEU251, ALA11, ASN248, LYS134, SER115, GLY114, ALA303, ASP282

## Data Availability

The data are contained within the article.

## Supplementary Materials

The following are available online at https://www.mdpi.com/article/10.3390/antiox14121500/s1.
