# Supplementary material for: Hepatoprotective, Antioxidant, and Anti-Hyperlipidemic Effects of Kefir Milk in High-Fat Diet-Induced Obesity: Insights from Gas Chromatography-Mass Spectrometry Profiling, Molecular Docking of Kefiran, and Liver Function Restoration
Source: Antioxidants (Basel). 2025 Dec 14;14(12):1500. doi: 10.3390/antiox14121500 (PMC12729788; doi:10.3390/antiox14121500)

Correlative Analysis

Correlative Analysis Between Hepatic and Testicular Tissues

A significant positive correlation was observed for several oxidative stress markers between liver and testis tissues in the context of high-fat diet (HFD)-induced obesity. MDA levels, an indicator of lipid peroxidation, showed a strong correlation ($r = 0.88$, $p < 0.05$), suggesting coordinated lipid oxidative processes across both tissues. Thiol groups, which play a key role in maintaining redox homeostasis, exhibited a strong correlation ($r = 0.79$, $p < 0.05$), indicating systemic regulation of antioxidant defenses.

Kefir milk (KM) supplementation notably reduced MDA levels and increased thiol concentrations in both liver and testis tissues, reflecting its potent antioxidant effects. These changes were more pronounced in KM-treated rats compared to the HFD-only group, highlighting KM's ability to mitigate oxidative stress induced by obesity.

Among the antioxidant enzymes, SOD levels displayed the highest correlation between the two tissues ($r = 0.928$, $p < 0.05$), reflecting a tightly coordinated systemic response to superoxide radical neutralization. CAT levels demonstrated a moderate yet significant correlation ($r = 0.78$, $p < 0.05$), suggesting partial interdependence in hydrogen peroxide detoxification pathways. In contrast, no significant correlation was found for GPx activity ($r = 0.08$, $p > 0.05$), implying a tissue-specific regulation of this enzyme. Importantly, KM administration significantly enhanced the activities of SOD, CAT, and GPx compared to the HFD-only group, further underscoring its hepatoprotective and antioxidant properties.

These findings highlight the role of kefir milk in restoring the oxidative balance disrupted by HFD-induced obesity, with coordinated improvements in systemic oxidative stress markers and tissue-specific regulation of antioxidant defenses.

GPx:

Correlations (Spreadsheet1) Marked correlations are significant at p < ,05000 N=24 (Casewise deletion of missing data)				
	**Means**	**Std.Dev.**	**GPx liver**	**GPx Testis**
**GPx liver**	26,54335	6,371281	1,000000	0,088462
**GPx Testis**	17,03577	2,433028	0,088462	1,000000

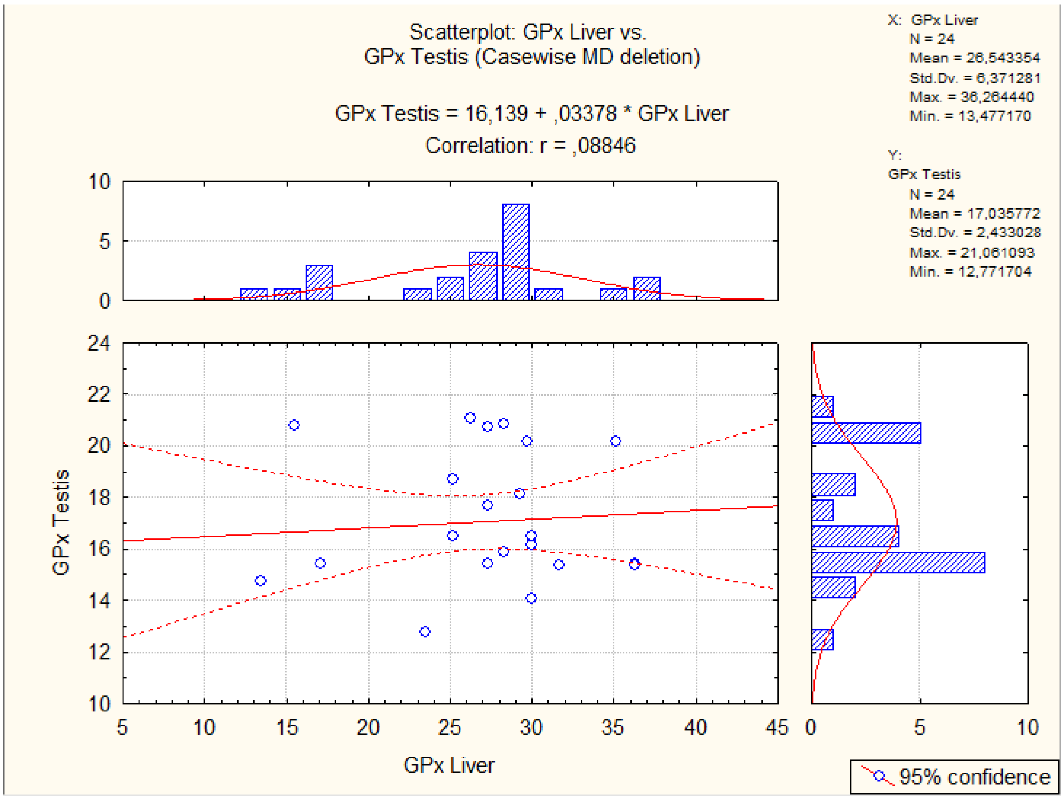

CAT

Correlations (Spreadsheet1) Marked correlations are significant at p < ,05000 N=24 (Casewise deletion of missing data)				
	**Means**	**Std.Dev.**	**CAT Liver**	**CAT testis**
**CAT Liver**	138,9333	41,50478	1,000000	0,780551
**CAT testis**	63,3917	13,09747	0,780551	1,000000

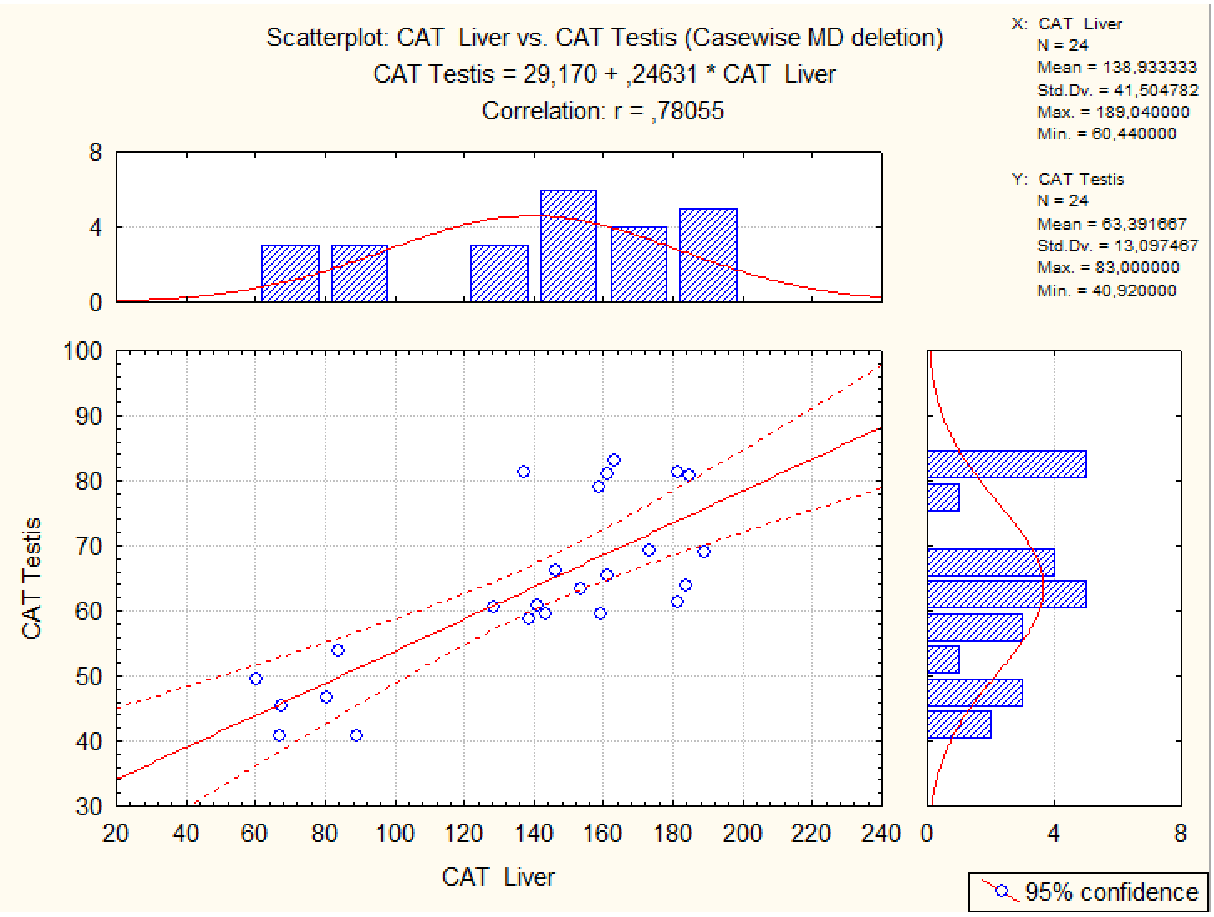

SOD

Correlations (Spreadsheet1) Marked correlations are significant at p < ,05000 N=24 (Casewise deletion of missing data)

	Means	Std.Dev.	SOD Liver	SOD testis
**SOD Liver**	2,714792	0,808704	1,000000	0,928172
**SOD testis**	1,403661	0,388701	0,928172	1,000000

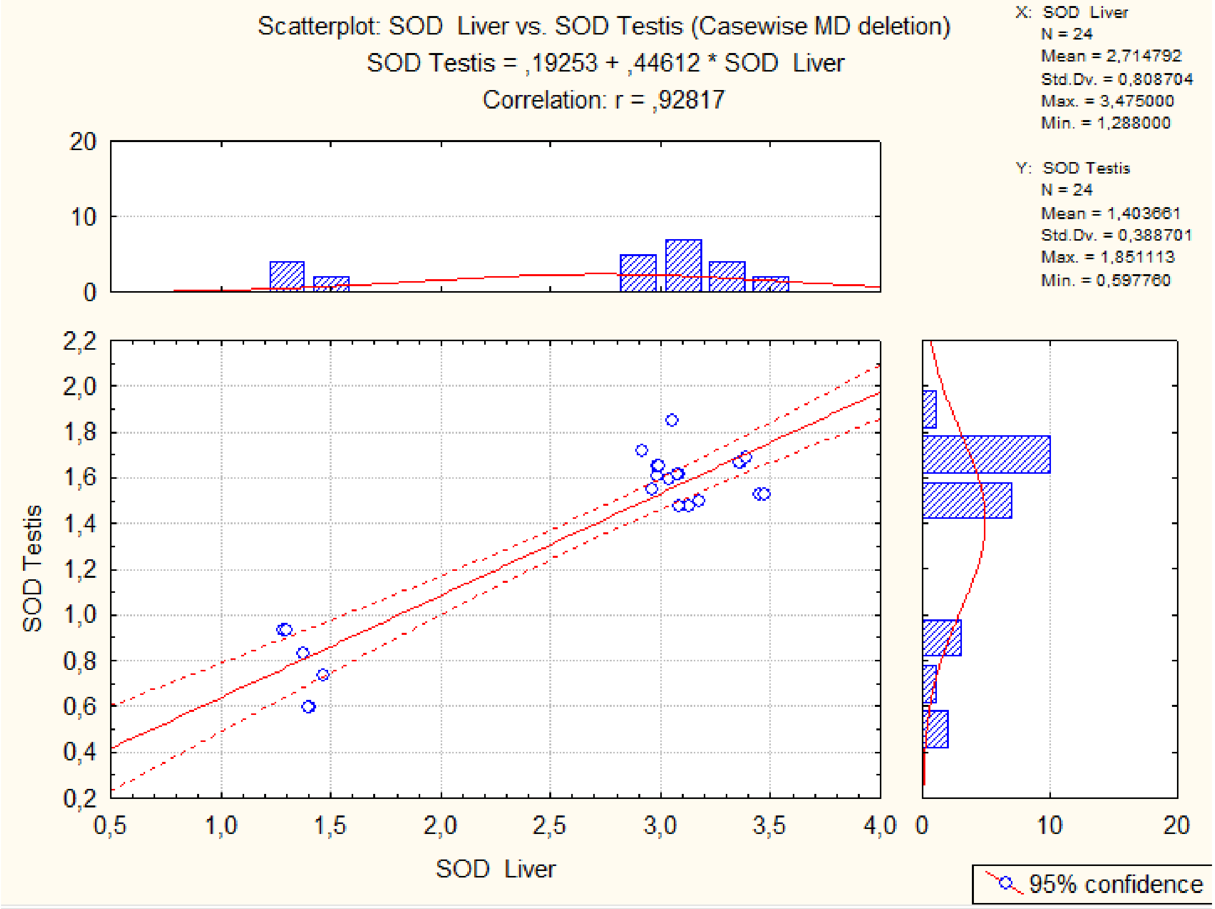

Thiols

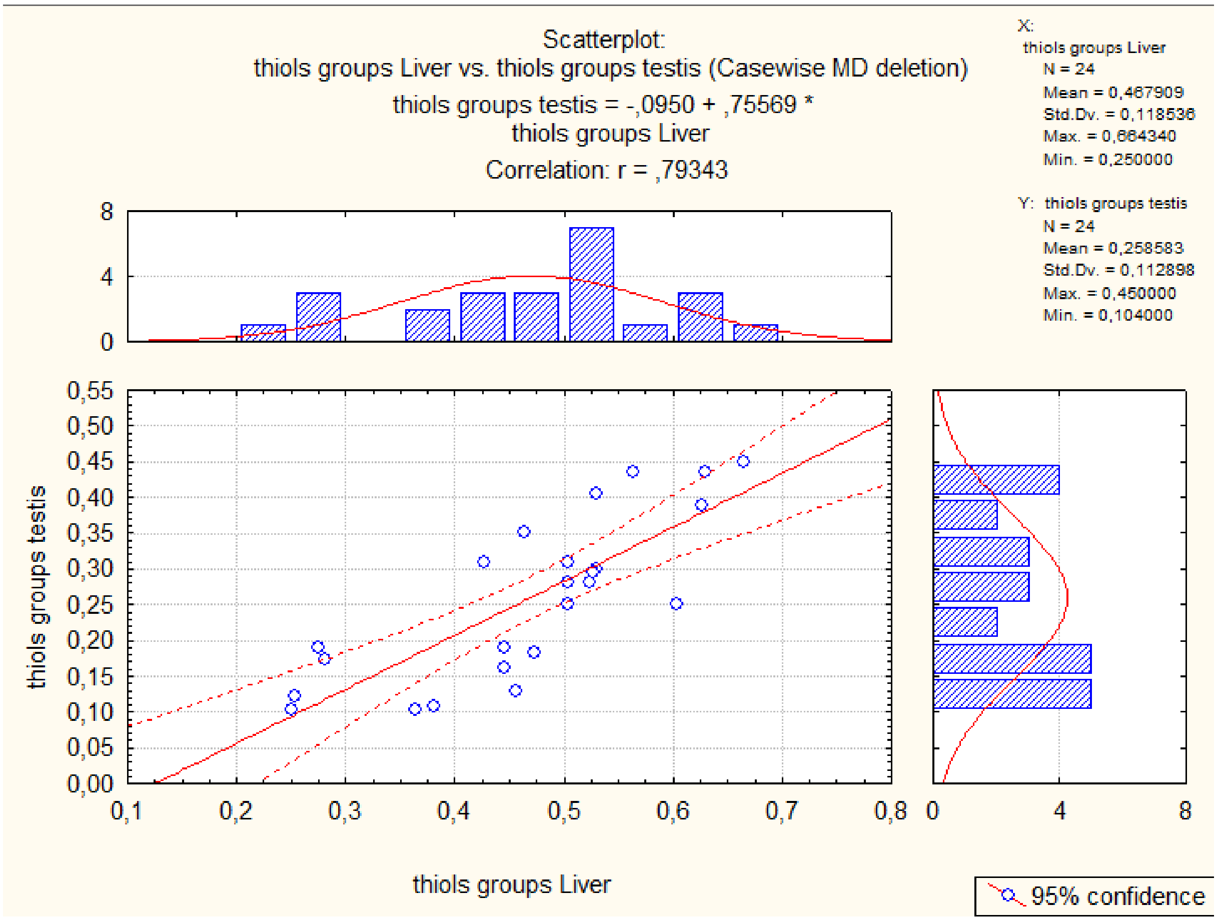

Scatterplot:
thiols groups Liver vs. thiols groups testis (Casewise MD deletion)
thiols groups testis = -,0950 + ,75569 *
thiols groups Liver
Correlation: r = ,79343
X:
thiols groups Liver
N = 24
Mean = 0,467909
Std.Dv. = 0,118536
Max. = 0,664340
Min. = 0,250000
Y: thiols groups testis
N = 24
Mean = 0,258583
Std.Dv. = 0,112898
Max. = 0,450000
Min. = 0,104000
thiols groups testis
thiols groups Liver
95% confidence

MDA

Correlations (Spreadsheet1) Marked correlations are significant at p < ,05000 N=24 (Casewise deletion of missing data)

	Means	Std.Dev.	MDA Liver	MDA testis
**MDA Liver**	0,283912	0,085259	1,000000	0,809865
**MDA testis**	0,213510	0,076984	0,809865	1,000000

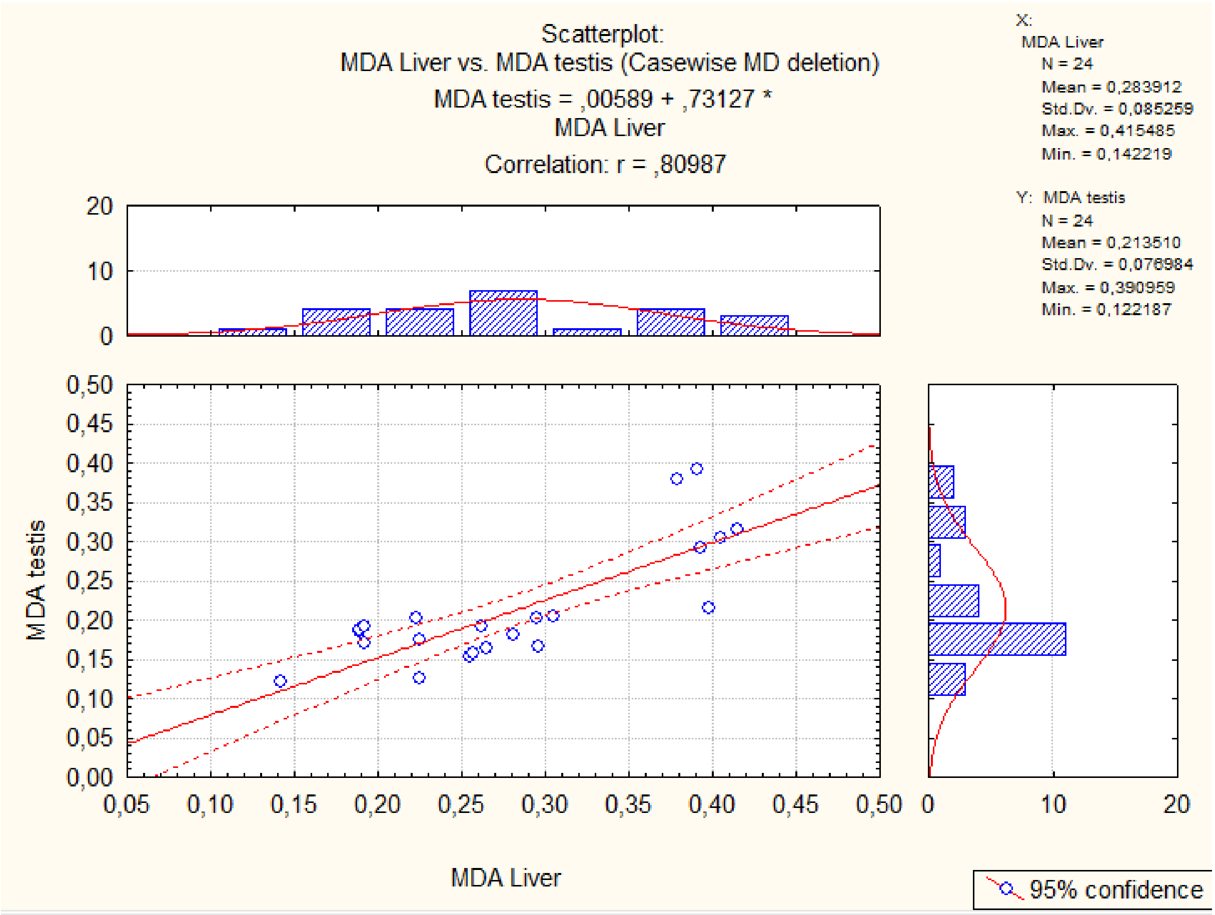

Supplement: Supplementary file 1 [file antioxidants-14-01500-s001.zip › antioxidants-3387175-supplementary.pdf]
